# Striatins as plaque molecules of *zonulae adhaerentes* in simple epithelia, of tessellate junctions in stratified epithelia, of cardiac composite junctions and of various size classes of lateral adherens junctions in cultures of epithelia- and carcinoma-derived cells

**DOI:** 10.1007/s00441-014-2053-z

**Published:** 2014-12-12

**Authors:** Werner W. Franke, Steffen Rickelt, Ralf Zimbelmann, Yvette Dörflinger, Caecilia Kuhn, Norbert Frey, Hans Heid, Rina Rosin-Arbesfeld

**Affiliations:** 1 Helmholtz Group for Cell Biology, German Cancer Research Center (DKFZ), Im Neuenheimer Feld 280, 69120 Heidelberg, Germany; 2 Progen Biotechnik, Heidelberg, Germany; 3 David H. Koch Institute for Integrative Cancer Research, Massachusetts Institute of Technology (MIT), Cambridge, Mass. USA; 4 Department of Internal Medicine III (Cardiology and Angiology), University Medical Center Schleswig-Holstein, Campus Kiel, Kiel, Germany; 5 Department of Clinical Microbiology and Immunology, Sackler School of Medicine, Tel-Aviv University, Tel-Aviv, Israel

**Keywords:** Adherens junctions, Tessellate junctions, Composite junctions, Intercalated disks, Arrhythmogenic ventricular cardiomyopathy (AC), Dilated cardiomyopathy (DC)

## Abstract

Proteins of the striatin family (striatins 1–4; sizes ranging from 90 to 110 kDa on SDS-polyacrylamide gel electrophoresis) are highly homologous in their amino acid sequences but can differ in their cell-type-specific gene expression patterns and biological functions. In various cell types, we have found one, two or three polypeptides of this evolutionarily old and nearly ubiquitous family of proteins known to serve as scaffold proteins for diverse protein complexes. Light and electron microscopic immunolocalization methods have revealed striatins in mammalian cell-cell adherens junctions (AJs). In simple epithelia, we have localized striatins as constitutive components of the plaques of the subapical *zonulae adhaerentes* of cells, including intestinal, glandular, ductal and urothelial cells and hepatocytes. Striatins colocalize with E-cadherin or E–N-cadherin heterodimers and with the plaque proteins α- and β-catenin, p120 and p0071. In some epithelia and carcinomas and in cultured cells derived therefrom, striatins are also seen in lateral AJs. In stratified epithelia and in corresponding squamous cell carcinomas, striatins can be found in plaques of some forms of tessellate junctions. Moreover, striatins are major plaque proteins of composite junctions (CJs; *areae compositae*) in the intercalated disks connecting cardiomyocytes, colocalizing with other CJ molecules, including plectin and ankyrin-G. We discuss the “multimodulator” scaffold roles of striatins in the initiation and regulation of the formation of various complex particles and structures. We propose that striatins are included in the diagnostic candidate list of proteins that, in the CJs of human hearts, can occur in mutated forms in the pathogeneses of hereditary cardiomyopathies, as seen in some types of genetically determined heart damage in boxer dogs.

**Electronic supplementary material:**

The online version of this article (doi:10.1007/s00441-014-2053-z) contains supplementary material, which is available to authorized users.

## Introduction

The adhering junctions, i.e. desmosomes (*maculae adhaerentes*) and adherens junctions (AJs), are major and important elements of the cell-cell connection system in tissues (Farquhar and Palade 1963; for a recent review, see Franke 2009). In the last two decades, the list of the various subtypes of these junctions has been extended by several cell-type-specific forms, including the *complexus adhaerens* of endothelial cells in certain types of lymph vessels (e.g. Schmelz and Franke 1990, 1993; Schmelz et al. 1994; for a review, see Moll et al. 2009) and the taproot junctions (*manubria adhaerentia*) of various mesenchymal cells (Wuchter et al. 2007). Of special physiological and medical interest are the myocardiac composite junctions (CJs; *areae compositae*) that form during the late fetal and postnatal heart development of diverse mammals and that represent dense-packed amalgamated arrays of molecules known as desmosomal, peridesmosomal and AJ components of simple epithelia (e.g. Borrmann et al. 2000, 2006; Franke et al. 2006, 2013, 2014; Pieperhoff and Franke 2007; Pieperhoff et al. 2008). The importance of this type of junction became evident from developmental studies of gene knock-out mice lacking plakoglobin or plakophilin-2 (Bierkamp et al. 1996; Ruiz et al. 1996; Grossmann et al. 2004) and from discoveries of specific, genetically determined cardiomyopathies in human and animal hearts (e.g. Gerull et al. 2004; Antoniades et al. 2006; Heuser et al. 2006; van Tintelen et al. 2006, 2007; Oxford et al. 2007a, b; Posch et al. 2008; Gehmlich et al. 2011; Gaertner et al. 2012; for the recent avalanche of literature, see reviews by Delmar and McKenna 2010; Murray 2012; Rickelt and Pieperhoff 2012; Patel and Green 2014).

In this context, a series of findings concerning hereditary cardiomyopathies in boxer dogs is remarkable; these have been reported to be based on a genetic predisposition for special forms of dilated cardiomyopathy (DC) or arrhythmogenic cardiomyopathy (AC). So far, this seems to be the only pathogenic situation known to involve mutations in a gene encoding a myocardiac member of the striatin family (“striatin mutations”; e.g. Meurs et al. 1999, 2007, 2010, 2013; Oxford et al. 2007a, 2011). Striatin 1 has repeatedly been reported to be specific for neural cells and functions (e.g. Castets et al. 1996, 2000; Bartoli et al. 1998, 1999; Kachidian et al. 1998; Salin et al. 1998; for other striatins, see also Muro et al. 1995; Moreno et al. 2000; for reviews, see Benoist et al. 2006; Hwang and Pallas 2013). On the other hand, Meurs et al. (2013) have claimed that myocardiac striatin is a desmosomal protein, whereas Breitman et al. (2008) have reported that striatins do not occur in desmosomes but in other kinds of junctions of epithelia and carcinoma cells (for a review, see Hwang and Pallas 2013). The elucidation and examination of possible pathogenic roles of mutated striatins is obviously necessary as these proteins are known as architectonic scaffold molecules able to form oligomers and complexes with other proteins, including kinases and phosphatases, calmodulin and specific Ca^2+^-binding proteins, cortactin-binding proteins and signal formation, transduction or vesicle translocation proteins (e.g. Muro et al. 1995; Kachidian et al. 1998; Salin et al. 1998; Bartoli et al. 1999; Moreno et al. 2000; Gaillard et al. 2001, 2006; Yu et al. 2001; Blondeau et al. 2003; Lu et al. 2004; Benoist et al. 2006; Goudreault et al. 2009; Gordon et al. 2011; Bobik 2012; Chen et al. 2012; Tanti et al. 2014; for a review, see Hwang and Pallas 2013).

As the members of the striatin family are highly homologous in their amino acid sequences, and as these isoforms and their splice variants can occur in cell-type-specific patterns, we have decided to address the family of striatin molecules in general in this report and will deal with the diverse cell-type-specific polypeptide isoforms, splice variants and biosynthesis details of striatins in a subsequent protein-chemical-oriented publication.

## Materials and methods

### Tissues and cell cultures

Bovine tissue samples were obtained from the regional slaughterhouse (Mannheim, Germany) and murine (rat and mouse) tissues were from animals of the laboratory-animal facilities of the German Cancer Research Center (Heidelberg, Germany; for details, see Franke et al. 2006). In addition, tissue specimens from fetal German landrace pigs and 3-year-old boars were obtained from the Institute of Farm Animal Genetics (Friedrich-Loeffler-Institute, Mariensee, Germany; see Rickelt et al. 2011). Cryopreserved human tissue samples, including tumour tissues, were obtained from material taken and examined for diagnostic pathology (Franke et al. 2006; Moll et al. 2009) or were provided by the National Center for Tumor Diseases (NCT, Heidelberg, Germany). In general, the samples were fixed either with 4 % formaldehyde in phosphate-buffered saline (PBS) and embedded in paraffin or were snap-frozen in isopentane that had been precooled in liquid nitrogen and were then stored at −80 °C until use. Protein lysates of frozen tissues were used for SDS-polyacrylamide gel electrophoresis (SDS-PAGE) of peptides (see Franke et al. 2013).

Monolayer cell cultures of various human cell lines were examined, including the breast-adenocarcinoma-derived line MCF-7, HaCaT keratinocytes, the colon-adenocarcinoma-derived lines CaCo2 and HT29 and the hepatocellular-carcinoma-derived cell lines PLC, HepG2, Hep3b and HuH7. Bovine epithelium-derived cell lines included mammary-gland-derived cells of lines BMGE, BMGE+H, BMGE+HE and KE-5. For comparison, rat liver hepatocellular carcinoma cells of the line MH1C1 were studied in parallel. The non-epithelial cell lines tested included the human cell lines U333/MG, K562, RPMI 8226, HL-60, SV80, WI-38 and RD, the bovine cell line B1, the rat cell line RVFSMC and the mouse cell lines 3 T3 and L929 (for further information, see Boda-Heggemann et al. 2009; Straub et al. 2011; Pieperhoff et al. 2012; Franke et al. 2013). In addition, freshly prepared cultures of human endothelial cells (HUVECs) and rat cardiomyocytes were used as described (cf. Pieperhoff et al. 2012).

### Antibodies

Primary monoclonal antibodies (mAbs) and guinea pig polyclonal antibodies (pAbs) were generated against several amino acid sequences (Table 1) of striatin family members obtained as polypeptides synthesized by PSL (Peptide Speciality Laboratories, Heidelberg, Germany). The peptides, coupled via cysteines to keyhole limpet haemocyanin (KLH), were used for the immunization of animals, in particular mice and guinea pigs. Further antibodies against proteins of the striatin family or other molecules used in biochemical and immunolocalization experiments are listed in the Electronic supplementary material (Table S1) and in the publication by Straub et al. (2003).

**Table 1:** Synthetic peptides (amino acid sequences) of striatin 1 used for coupling to keyhole limpet haemocyanin (*KLH*) protein and antibody production in guinea pigs (*NT* aminoterminal sequence, *CT* carboxyterminal sequence, *h* human). The same KLH-coupled peptides were used in a second immunization series. The *dot* in the aminoterminal sequence (N ∙ P) stands for a histidine residue left out at this position in the antigenic peptide. “Striatin mix” (see text) is a mixture of equal portions of all four antisera

Name	Amino acid sequence	Amino acid (aa) numbers
Striatin-hNT	MDEQAGPGVFFSNN ∙ P-C-KLH	aa 1-16
Striatin-h268	RKKALPDSGEDRD-C-KLH	aa 268-280
Striatin-h301	SRSAGDGTDWEKEDQ-C-KLH	aa 301-316
Striatin family-hCT	KLH-C-YIASAGADALAKVFV	aa 765-780

The protocols in which murine mAbs, guinea pig pAbs and other antibodies were used for immunofluorescence microscopy or for immunoblotting analyses of PAGE-separated polypeptides against AJ molecules or against diverse cytoskeletal proteins were as described elsewhere (Rickelt et al. 2011). The newly generated mAbs and pAbs were routinely compared with “anti-striatin” and “anti-SG2NA” mAbs purchased from Becton-Dickinson (Heidelberg, Germany) or Millipore (Temecula, Calif., USA) and with commercially available polyclonal rabbit antibodies against striatin 4 (“zinedin”; Acris Antibodies, Herford, Germany). Antigen-bound primary Abs were visualized with secondary antibodies coupled to Cy3 (Dianova, Hamburg, Germany) or Alexa 488 (MoBiTec, Göttingen, Germany). For immunoblot analysis, horseradish-peroxidase-conjugated secondary antibodies were applied (Dianova).

### Gel electrophoresis and immunoblotting

Protein lysates were analysed by SDS-PAGE, followed by immunoblotting, as described (Rickelt et al. 2011; Pieperhoff et al. 2012; Franke et al. 2013).

### Immunofluorescence and immunoelectron microscopy

Methods for immunofluorescence and electron microscopy were as previously described (Franke et al. 2006, 2013; Rickelt et al. 2011; Pieperhoff et al. 2012; Rickelt 2012).

## Results

### Characterization of striatin proteins and antibodies

At least three genes encoding striatins of highly homologous amino acid sequences (striatins 1, 3, 4) have been identified, each with a series of introns. These genes and introns can result in different cell-type expression patterns of the various isoforms and splice variants. In the present report, we have therefore tried to generate certain polypeptide-sequence-specific antibodies, including some that are specific for certain unique sequence epitopes and others that cross-react between different striatins (see Materials and methods, Table 1).

**Fig. 1:**
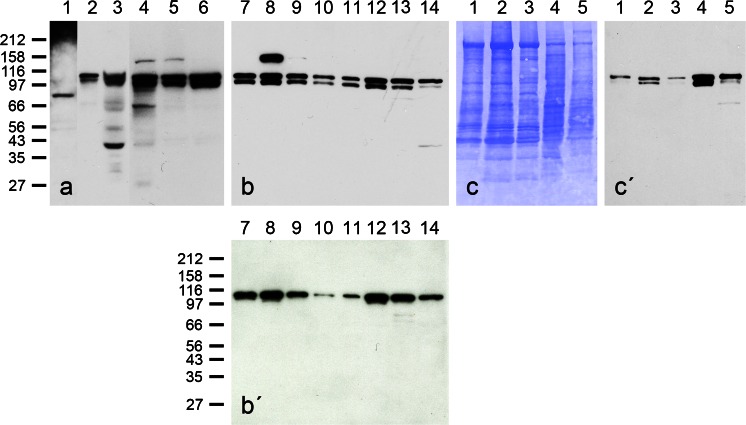
Results of SDS-polyacrylamide gel electrophoresis (SDS-PAGE)-separated polypeptides as obtained by immunoblot reactions (**a**, **b**, **b’**, **c’**) or Coomassie blue staining (**c**). The antibodies used were monoclonal antibody (mAb) “Striatin” (Becton-Dickinson; **a**, **b**, **c’**) and polyclonal antibody (pAb) raised in guinea pig (gp), namely “striatin mix” of NTB, 268B, 301B and CTB (**b’**). The tissue and cell lysis protein preparations used were from human heart (*lane 1*), tongue mucosa (*lane 2*), liver tissue (*lane 3*) and the following human cell culture lines: PLC (*lane 4*), HaCaT (*lane 5*), SV80 (*lane 6*), A498 (*lane 7*), CaCo2 (*lane 8*), A431 (*lane 9*), HeLa (*lane 10*), HUVEC cells (secondary cell culture; *lane 11*), K562 culture 01 (*lane 12*), K562 culture 02 (*lane 13*) and RPMI 8226 culture 01 (*lane 14*). In the results shown in **c** and **c’**, the following materials were used: human heart tissue (*lane 1*), bovine heart tissue (*lane 2*), murine heart tissue (mouse; *lane 3*), murine HL-1 culture line of cardiomyocytes (*lane 4*), and a primary cell culture of neonatal rat cardiomyocytes (*lane 5*). Note the dominant immunoblot polypeptide band at approximately 110 kDa in all tissues and cell cultures, except for the weak reaction in *lane 1* of **a**, which is, however, more noticeable at higher protein loadings. In addition, an immunoreactive band at approximately 100 kDa is seen in most lanes of **a**, **b** and in *lanes 2*, *4* of **c’**. In specific lanes of **a** and in *lane 8* of **b**, additional bands are notable that have not yet been characterized. Further reaction bands are seen at approximately 100 kDa (**a**, **b**, *lanes 2*, *4* in **c**) and at approximately 142 kDa (*lanes 4*, *5* in **a**, *lane 8* in **b**)

 Using the above antibodies and several that were commercially available, we identified striatins in all normal and tumour cells examined, including single blood cells and tissue cells and in cultured cells and tumour cells (Fig. 1a, b). Whereas some of these antibodies revealed the presence of at least two polypeptide bands of approximately 110 and 100 kDa (Fig. 1a), other sequence-specific antibodies reacted with only one polypeptide (cf. Fig. 1b, b’). The common bands identified by some of the antibodies often appeared rather faint on some tissues, notably those of liver and heart, but were much more intense at higher protein loads or after extended immunoblot exposure times (see also Electronic supplementary material, Fig. S1). When various preparations of mammalian heart tissue or murine cardiomyocyte cell cultures were compared, a band with an M_r_ of approximately 110 kDa was always seen and, in some preparations, was accompanied by a (mostly minor) band of a lower M_r_ (the obvious difference with respect to the SDS-PAGE immunoblot data of Castets et al. 2000, who reported only cardiac polypeptides of lower M_r_ values, i.e. approximately 94 and 100 kDa, cannot yet be explained).

### Colocalization experiments

Because of the dense-packing of cytoskeletal and cell junction components, special and carefully controlled antibody binding and differential washing protocols are needed to distinguish true and specific epitope binding from the various forms of structure and protein “stickiness”. In the present study, we have generally included diverse washing steps to remove non-specifically bound (“sticky”) material from the structures in question, and in a series of cases, this required brief (5, 10 or 15 min) rinsing with mild detergent-containing buffers and/or acetone solutions. To illustrate the importance of such differential washing steps, we include here, as an example, the binding and release reactions of desmoplakin and plectin (Electronic Supplementary Material, Fig. S2). Whereas desmoplakin is known as an extraction-resistant, intensely binding component of the CJs in the intercalated disks of the myocardium, plectin is, in addition, known for its marked “sticky” behaviour, i.e. binding that is not immunologically determined. Consequently, the plectin reaction with the sarcomeric Z-lines can (and should) be removed by differential washing (Fig. S2; cf. Fig. S2a, b). On the other hand, in the course of these washing steps, a significant portion of the plectin antibodies remain bound to the CJs, thus resulting, together with established CJ markers, such as desmoplakin, in a typical yellow merged reaction colour (Fig. S2a’’, b’’). For example, the differential localization reaction of a “sticky” protein and a CJ-specific plaque protein is shown in Fig. S3, comparing striatin as a CJ-specific protein with α-actinin as a “sticky” sarcomeric Z-line protein (Fig. S3; cf. Bennett et al. 2006). By contrast, various types of antibodies against α-actinin colocalize with high precision and intensity (see the yellow merged patttern in Fig. S4). Consequently, extensive differential washing treatments have been included in the immunolocalization experiments of this study.

### Light microscopic immunolocalization in simple epithelial tissues

**Fig. 2:**
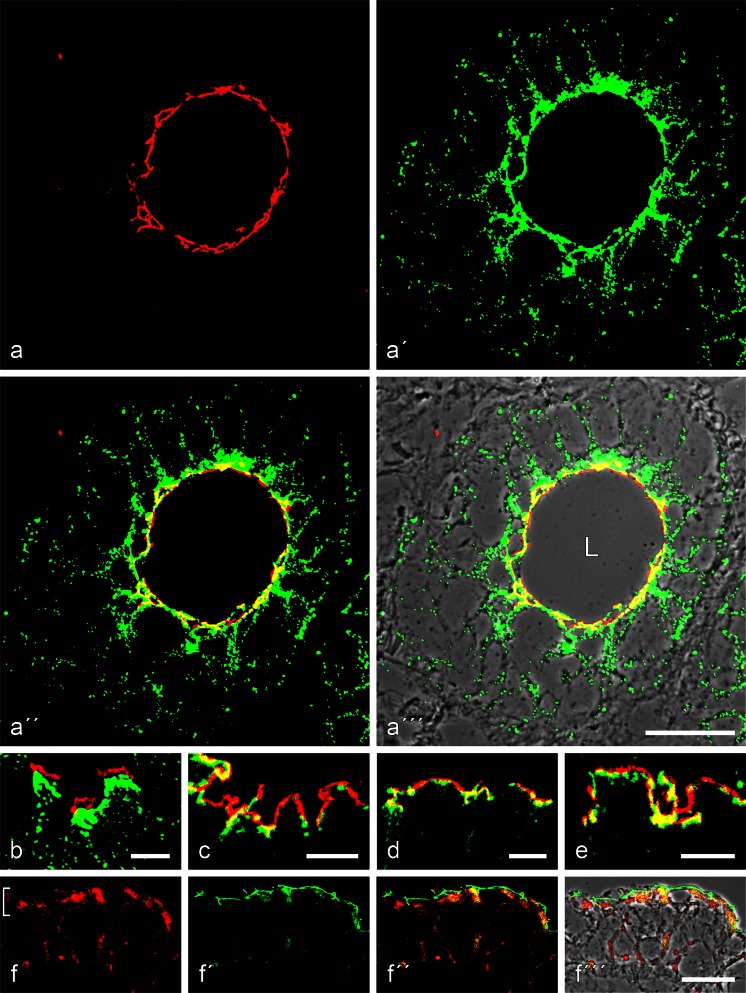
Immunolocalization of striatin in the *zonula adhaerens* of bovine intestinal epithelial cells. **a–a’’’** Double-label confocal laser scanning immunofluorescence microscopy, showing striatin (**a**, *red*; mAb mouse [m]) in a relatively narrow apical zone in partial colour reaction overlap with desmoplakin (**a’**, *green*; pAb gp). This double-label immunoreaction is seen in **a’’** and on a phase contrast background in **a’’’**. Note, however, that striatin is restricted to the subapical ring (*zonula adhaerens*; *L*, *lumen*), whereas desmoplakin is also located in the numerous desmosomes of the basolateral cell-cell contacts. **b** Higher resolution micrograph showing the distinct separation of the striatin-positive *zonula* (*red*) and the basolateral desmosomes (*green*). **c–e** Differential immunostaining reactions of striatin (*red*; mAb m) and β-catenin (*green*; rabbit [rb] antibody), indicative of zones of colocalization (*yellow*) and local segregation (*red*). **f–f’’’** Double-label immunofluorescence microscopy showing the same tissue after localization of proteins PERP (**f**, **f’’**, **f’’’**, *red*; mAb m) and striatin (**f’–f’’’**, *green*; gp). Note that striatin is restricted to a thin upper line of the *zonula adhaerens* (*green*), whereas protein PERP is seen in a slightly lower zone and in special larger punctate structures both at the *zonula* and at the basolateral membranes (*bracket* and *bottom* in **f**, respectively). *Bars* 20 μm (**a**, **f**), 5 μm (**b–e**)

**Fig. 3:**
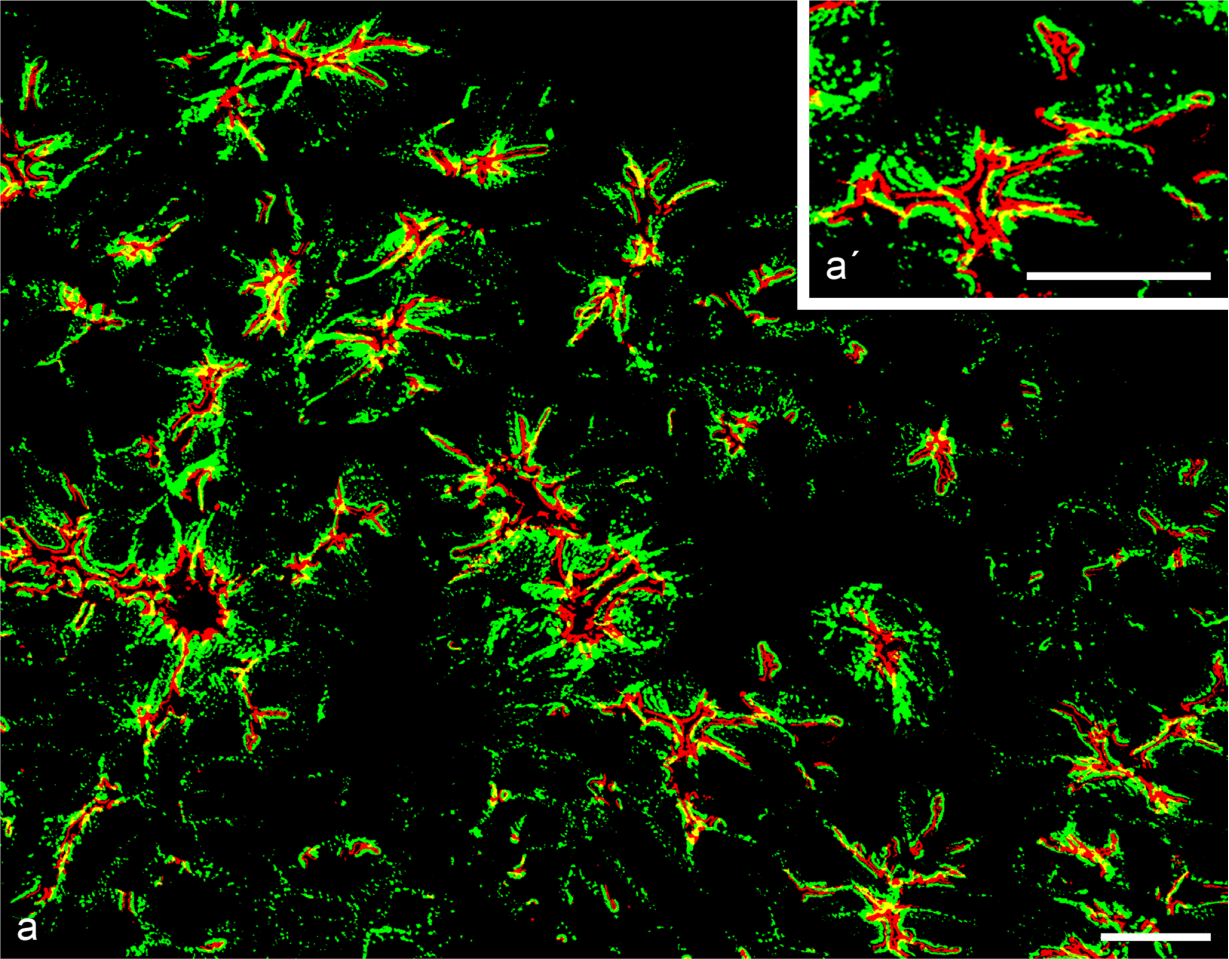
Double-label confocal laser-scanning immunofluorescence microscopy of cryostat sections through a bovine muzzle epidermis region rich in glandular and ductal epithelium. **a** Survey of cross- or obliquely-sectioned gland structures showing the general frequency of desmosomes (*green*; desmoplakin, gp antibody) and *zonulae adhaerentes* positive for striatin (*red*; mAb m). **a’** Higher magnification illustrating the differential localization of these two structures. *Bars* 20 μm

Using various antibodies against members of the striatin family on cryostat sections of diverse forms of simple epithelia, we have obtained a distinct immunolocalization pattern marking the upper portion in the *zonula adhaerens*. Figure 2a-a’’’, for example, presents bovine intestinal epithelium. At the limited resolution in such semithin sections, the striatin reaction, for the most part, overlaps with that of the apical-most desmosomes (yellow merger colour), whereas the desmosomes lying more basally on the lateral cell membranes do not react at all or overlap optically with striatin antibodies (e.g. Fig. 2b). In thin sections, however, one can even locally often distinguish a specific thin apical striatin-positive *zonula*-like structure from the subjacent general *zonula adhaerens* region reacting, for example, with α-catenin, β-catenin, p120, p0071 and protein ZO-1 (for β-catenin, see Fig. 2c–e). In such thin sections, the striatin and β-cadherin-positive zones can also be distinguished from the adjacent occludin- and claudin-positive *zonulae occludentes* of tight junctions (TJ; not shown). This subapical *zonula* region is also different from the *zonula* reaction sites of the 21-kDa transmembrane protein PERP (Fig. 2’’’; the apical zone under discussion here is demarcated by the bracket symbol in Fig. 2b). Similar results have been obtained in diverse tissues with adluminal simple epithelia, including salivary and other glandular epithelia, duct epithelia and pulmonary epithelium, bladder urothelium and the seminiferous and excurrent duct epithelia of the testis (cf. Domke et al. 2014). In bovine muzzle epithelial glands and ducts (Fig. 3), the marked striatin *zonula* immunostaining is seen in both the secretory and the ductal cells. Essentially identical results have been obtained in all five mammalian species examined.

**Fig. 4:**
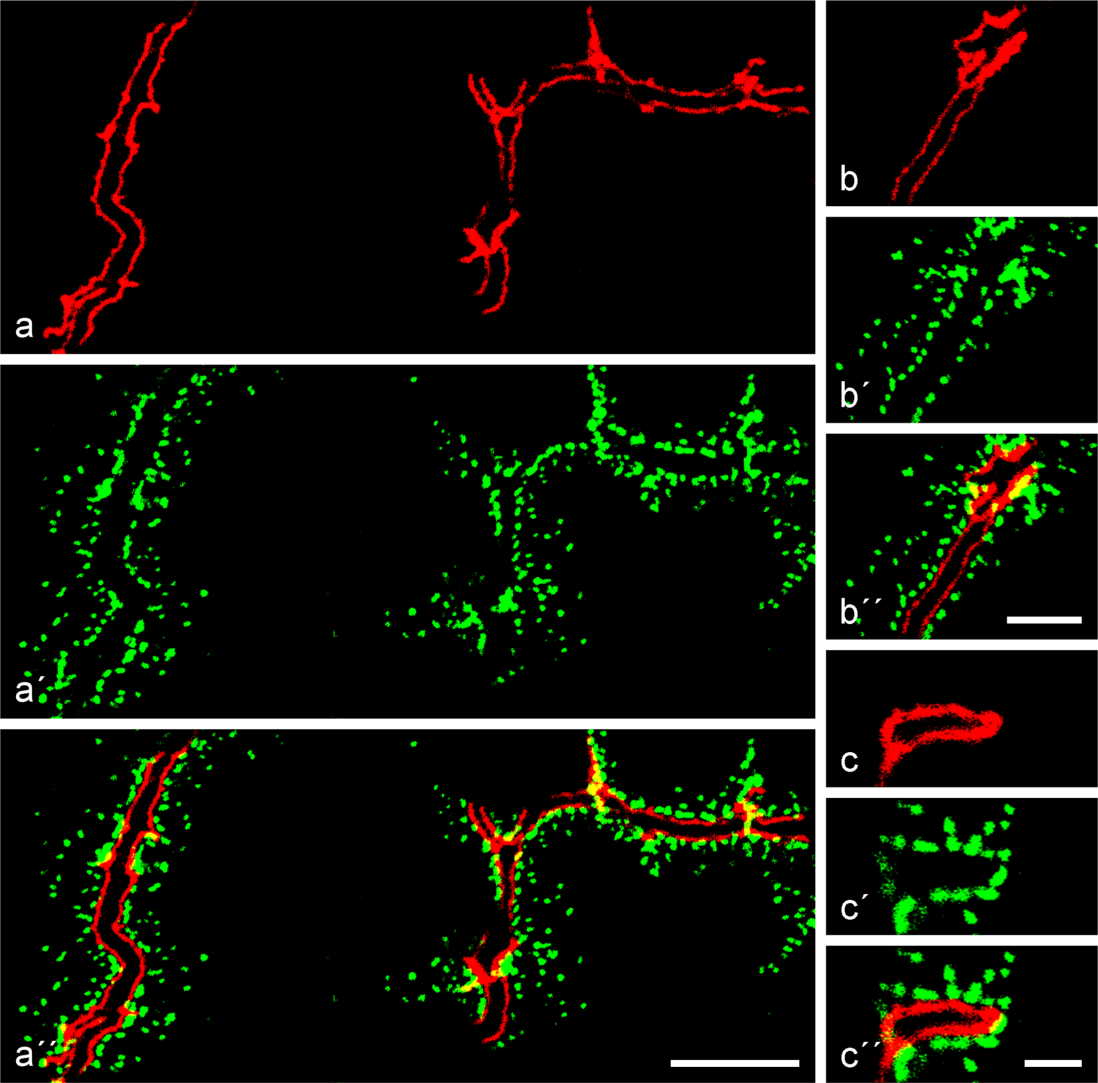
Specific immunolocalization of striatin in the *zonula adhaerens* structures of hepatocytes extending along the bile canalicular surface in bovine liver tissue. **a–a’’** Double-label confocal laser scanning immunofluorescence microscopy of near-longitudinal sections of bile canaliculi, showing the location of striatin (**a**, *red*; mAb m) in comparison with the strictly desmosomal position of desmoplakin (**a’**, *green*; pAb gp). Note that in double-colour labelling (**a’’**), the reaction of “free” desmosomes is strictly *green*, indicating that the desmosomes do not contain any striatin. **b–b’’** Same preparation as in **a–a’’** showing that, for most of the section, an unstained space of the canalicular lumen is seen between the striatin-rich layer of the *zonula* (*red*) and the desmoplakin-positive desmosomes (**b’**, **b’’**, *green*). **c–c’’** Same preparation showing a near exact cross-section through a bile canaliculus surrounded by a continuous striatin-positive *zonula* (*red*) and a series of desmoplakin-positive desmosomes (**c’**, **c’’**, *green*). *Bars* 10 μm (**a**), 5 μm (**b**), 2.5 μm (**c**)

As the polar organization of the hepatocytes in mammalian liver tissue represents an especially complex junction, and as liver physiology and diseases are of special importance, we have performed detailed double- and triple-label high-resolution immunolocalization reactions on cryostat liver sections of the five mammalian species used, namely mouse, rat, pig, cattle and human. Figure 4 presents the results obtained by double-label immunofluorescence microscopy, comparing the punctate, rather regularly spaced desmoplakin reaction sites along the bile canaliculi with the thin and distinct, but also intensive, striatin reaction of the apical *zonula adhaerens* structures (Fig. 4a-a’’, b-b’’). Moreover in cross-sections through the bile canaliculi, we could demonstrate (Fig. 4c-c’’) the entire subapical plasma membrane reaction of striatin in direct comparison with the surrounding desmosomes. These results were identical in all five species and were also similar to those obtained for other *zonula adhaerens* markers, including the proteins myozap (Rickelt et al. 2011) and LUMA (Franke et al. (2014).

### Light microscopic immunolocalization in stratified epithelia

Epithelia of this category are characterized by variously sized and variously structured interdesmosomal regions that can be studded not only with “gap junctions”, but also with single molecules or “islands” of TJ and/or AJ molecules (“tessellate junctions”; cf. Franke and Pape 2012; Franke et al. 2013). The patterns of these interdesmosomal cell-cell junction structures vary markedly not only between the various types of epithelia, but also in the various cell layers.

**Fig. 5:**
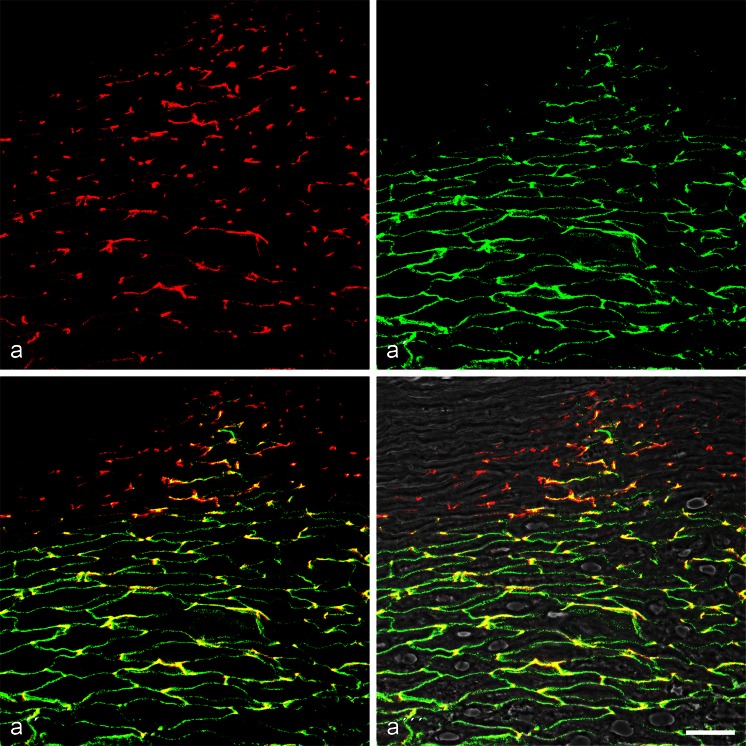
Double-label confocal laser scanning immunofluorescence microscopy showing one of the subforms of cell-cell tessellate junctions in a multistratified epithelium, namely the ventral part of bovine tongue mucosa. **a–a’’’** Striatin (*red*; mAb m) demonstration in regional substructures, including polar or *fascia*-like tessellate junctions; the β-catenin-positive portion (*green*; pAb gp) of the tessellate junctions extends over much larger cell-cell contact areas. *Bar* 20 μm

Punctate and *fascia*-like striatin immunolocalization reactions have also been noted in the various stratified epithelia examined. For example, the distribution of striatin-containing portions in tessellate junction layers of bovine tongue mucosa is shown in comparison with immunostaining for β-catenin in Fig. 5. In the interdesmosomal cell-cell contact regions of these stratified tissues, small punctate *fascia*-like or even more extended striatin reaction sites are often seen, mostly showing colocalization of AJ molecules with TJ markers such as occludin, because of spatial overlap. Moreover, in several stratified epithelia, striatin immunostaining is not restricted to colocalization areas with other AJ proteins but has selectively been noted in upper layers, positionally equivalent to the upper *stratum spinosum* and the *granulosum* layers of the epidermis, even in regions that appear totally negative for proteins including the catenins, p120, p0071 and protein ZO-1 (see Fig. 5, upper portion). The reactive structures and the intensities of the various members of the striatin protein family can differ in the different stratified epithelia, i.e. epidermis, oral and lingual mucosa regions, oesophagus, pharynx epithelium and stratified thymic reticulum epithelium (“Hassall bodies”). Therefore, we have decided to devote a special future article to the complex patterns of AJ protein localizations in the distinct substructures of tessellate junctions of mammalian stratified epithelia and in tumours and cell cultures derived therefrom.

### Immunolocalization in myocardiac tissues

In view of the molecular architectonic, functional and medical importance of CJs in the intercalated disks, and in view of the special roles of such molecules in the pathogenesis of a series of heart diseases and “sudden death” forms, we have carefully examined and compared the five mammalian species mentioned by immunofluorescence microscopy. Moreover, as a single striatin polypeptide appeared to be the predominant, if not exclusive isoform in myocardiac cells in situ (see Fig. 1a, c’), we made certain, in all cases, that the antibodies specific for this striatin were included in the experiments.

**Fig. 6:**
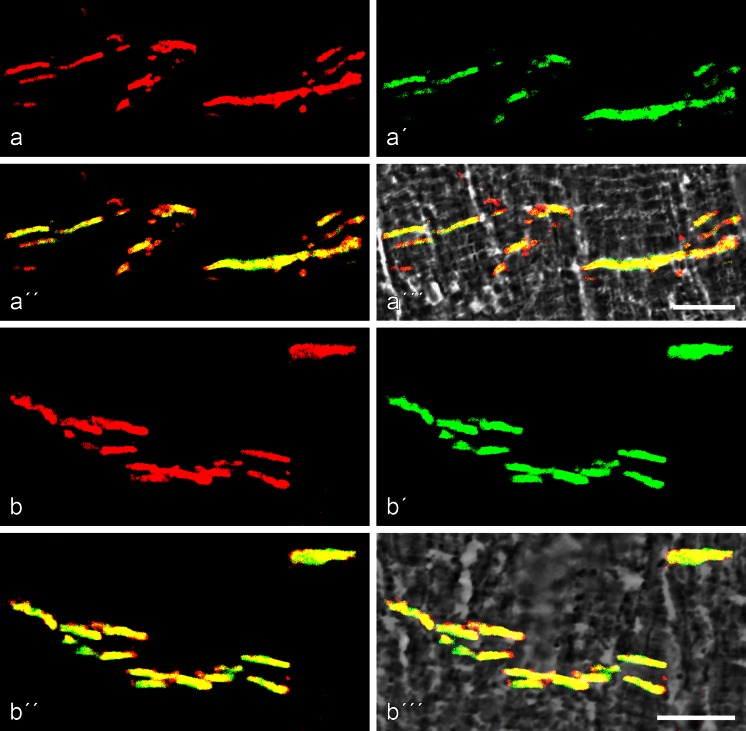
Double-label confocal laser scanning immunofluorescence microscopy of cryostat sections through porcine (boar) myocardium. **a–a’’’** Immunostaining of striatin in the complete composite junctions (*areae compositae*) of the intercalated disks (**a’**, *green*; pAb gp) in comparison with β-catenin (**a**, *red*, mAb m), showing extensive colocalization (**a’’**, *yellow* merged colour; **a’’’**, as **a’’**, but with a phase contrast background). **b–b’’’** Parallel preparation to that of **a–a’’’** but after reactions with antibodies to N-cadherin (**b**, *red*; mAb m) and to striatin (**b’**, *green*; pAb, gp), again showing colocalization (*yellow* merged colour) without (**b’’**) and with (**b’’’**) phase contrast background. *Bars* 10 μm

**Fig. 7:**
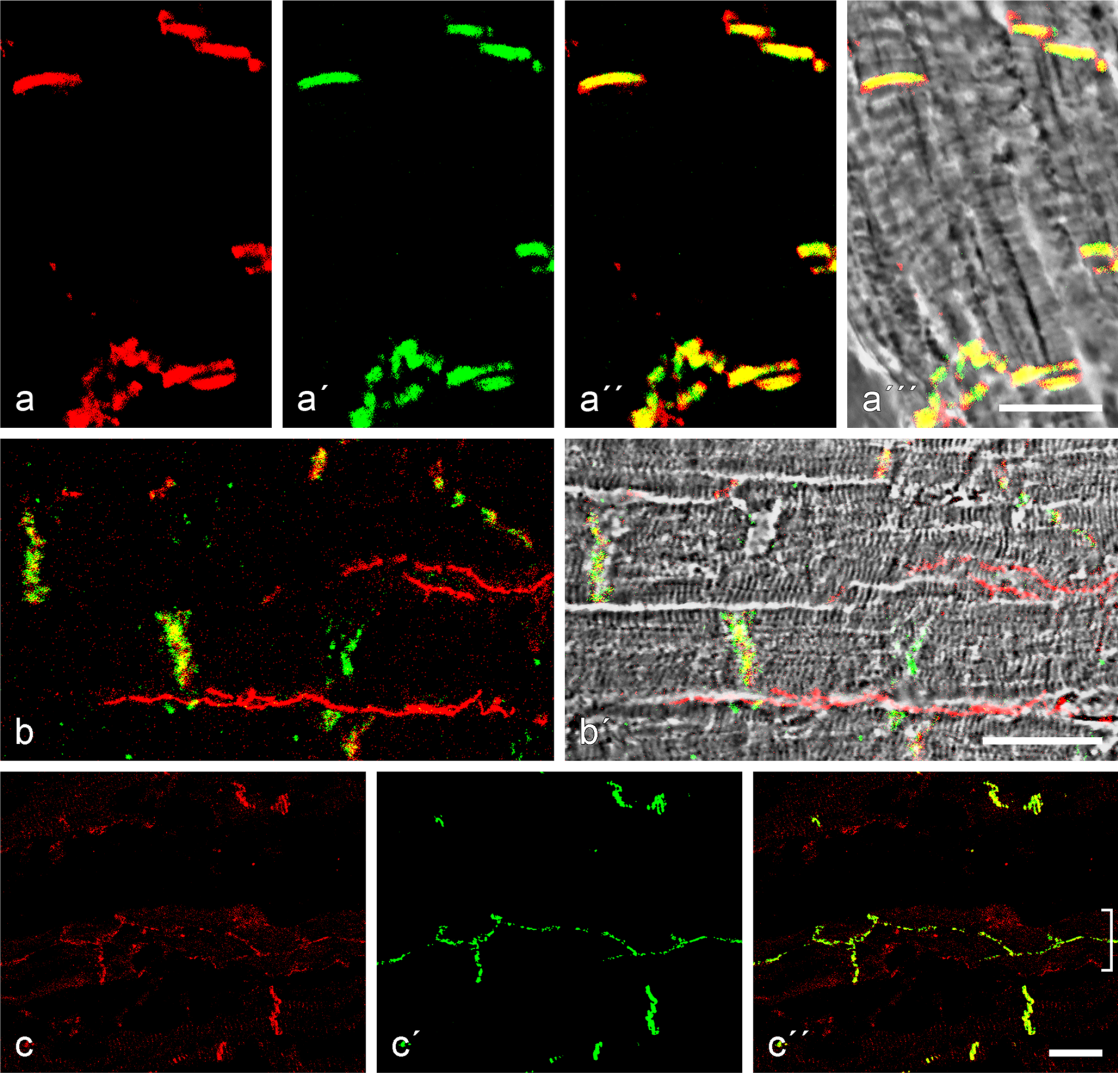
Double-label confocal laser scanning immunofluorescence microscopy of cryostat sections through bovine myocardium. **a–a’’’** Immunostaining of desmoplakin (**a**, *red*, mAb m) compared with that of striatin (**a’**, *green*; pAb gp) shows extensive colocalization (*yellow* merged colour in **a’’** without and in **a’’’** with phase contrast background). Note also some small but distinct punctate staining regions that are either *red*, i.e. positive for desmoplakin, or *green*, i.e. positive for striatin. **b**, **b’** Double-labelling with antibodies to the plaque protein ZO-1 (**b**, *red*; mAb m) in comparison with antibodies to striatin (**b**, *green*; pAb gp) shows colocalization in the composite junctions but exclusive protein ZO-1 labelling at the junctions of the cardiac capillary endothelium (*red*). The relationship to the specific structures is seen in the phase contrast backbground image (**b’**). Note also some small strictly *red* or *green* punctate structures. **c–c’’** Double-labelling immunostaining of striatin (**c**, *red*; mAb m) and desmoplakin (**c’**, *green*; pAb gp) in **c’’** shows that the conductive cells of the Purkinje cell system also contain some junctions that appear *yellow* (merged colour in **c''**, *bracket*). *Bars* 20 μm

As shown for the example of boar heart (Fig. 6), striatin is highly enriched in the CJs, usually showing colocalization with N-cadherin and β-catenin (Fig. 6a-a’’’, b-b’’’) and with p120, desmoplakin, plakophilin-2 and desmoglein-2 (Dsg2; not shown). Essentially identical colocalization results have been obtained for bovine cardiomyocytes in situ (Fig. 7a-a’’’ presents, for example, colocalization with desmoplakin). Several other AJ plaque proteins such as ZO-1 also colocalize with striatin in CJs but here striatin label has not been detected in the *zonulae adhaerentes* of the interspersed blood capillaries (Fig. 7b, b’).

Colocalization of striatin with other CJ molecules has also been found in a significant proportion of the junctions of the Purkinje network of conducting cells (Fig. 7c-c’’; for an extensive recent review of cardiac conduction cells, see Mezzano et al. 2014). In addition, we have noted, in the conductive cells, a few striatin-positive junctional reaction sites that are negative for all desmosome-specific marker proteins and other junctions that are desmoplakin- and plakophilin-2-positive but negative for all striatin antibodies tested (not shown).

**Fig. 8:**
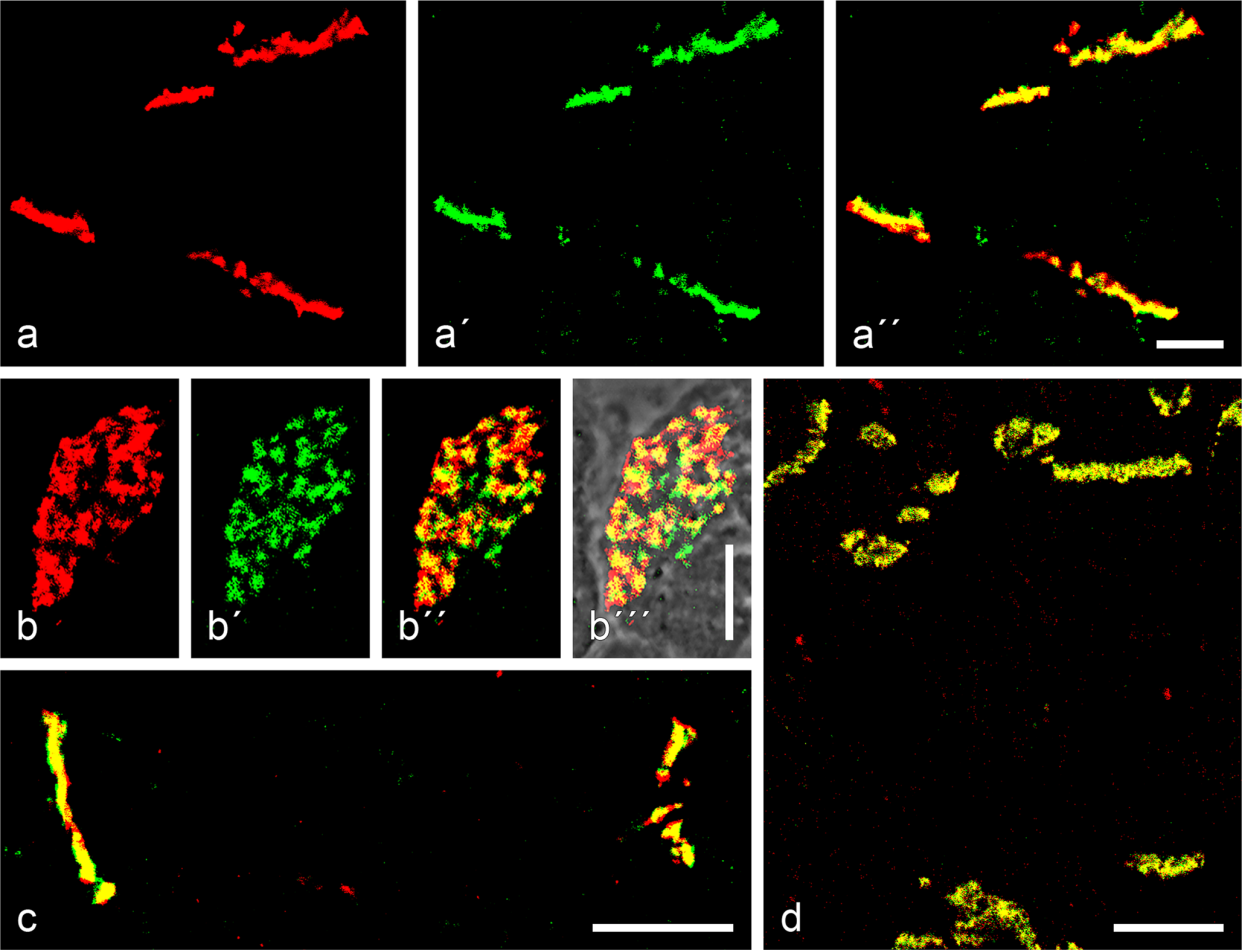
Double-label confocal laser scanning immunofluorescence microscopy of cryostat sections through human myocardium. **a–a’’** Colocalization of N-cadherin (**a**, *red*; mAb m) and striatin (**a’**, *green*; pAb gp) on cross-sections of composite junctions (**a’’**, *yellow* merged colour). Note, however, the occurrence of some small isolated punctate striatin-positive, i.e. *green*, reaction sites (e.g. in the middle between the two composite junctions in the lower part of the image). **b–b’’’** Colocalization (reagents as in **a–a’’**) of N-cadherin (**b**) and striatin (**b’**) on near-horizontal sections of intercalated disks (**b’’**, **b’’’** merged images, presented in **b’’’** on a phase contrast background). Note the *yellow* merged colour in each of the composite junction substructures. **c** Colocalization of striatin (*green*; pAb gp) with desmoplakin (*red*; mAb m) in the plaques of composite junctions. Note also a tiny punctate and exclusively desmoplakin-positive reaction site (*middle bottom*). **d** Control of the colocalization approach by using two different antibodies reactive with striatin: mAb (m) reactive with different striatins (*red*) and pAb (gp) reactive only with a specific striatin type (*green*). The colocalization (*yellow* merged colour) shows that striatin3 is predominant and that no other striatin is recognized in the myocardium (note the tiny "artificial" *red dot* in the centre of the region *right* serving as a control). *Bars* 10 μm (**a**, **b**), 20 μm (**c**, **d**)

In both murine species examined, the colocalization of striatin with β-catenin (Fig. 8a-a’’’), p120 and p0071 and with desmoglein-2, desmoplakin, plakophilin-2 and plakoglobin have also been observed. However, in a number of experiments, we have also detected small (“dot-like”) cytoplasmic reaction sites that are positive only for striatin or only for specific CJ partner molecules such as β-catenin (see Electronic supplementary material, Fig. S5), p120 and p0071 or α-catenin (not shown).

Unsurprisingly, the same kind of results were also obtained in our extensive localization studies of human myocardium. As shown in cross-sections of intercalated disks (Fig. 8a-a’’, c) and in grazing-horizontal sections of intercalated disks (Fig. 8b–b’’’), pronounced colocalization was typical for at least one isoform of striatins with N-cadherin (Fig. 8a–a’’’, b-b’’’), β-catenin, p120 and p0071 (not shown) and with desmoplakin (Fig. 8c), desmoglein Dsg2, plakophilin-2 and plakoglobin (not shown). In special control experiments, we also used two different types of striatin antibodies, namely, one that cross-reacted with various striatins and one that reacted only with the major cardiac striatin polypeptide. Again, near-complete colocalization was observed (Fig. 8d).

Colocalization of striatin(s) in the plaques of CJs has also been found for plectin, an extremely large protein previously described in association with various other contractile and cytoskeletal proteins (Wiche et al. 1983; Wiche 1989; Andrä et al. 1997; for biochemical data, see also Wiche et al. 1982; Pieperhoff et al. 2012) and ankyrin-G (Electronic supplementary material, Fig. S6), confirming the data of Makara et al. (2014; see also Mohler et al. 2004; Sato et al. 2011; for a review, see Bennett and Healy 2009). To demonstrate the specificity and intensity of the binding of ankyrin-G and plectin to other CJ plaque proteins and, notably, also to the protein myozap (see also Pieperhoff et al. 2012) and striatin (Fig. S6f), gradual “buffer wash treatments” of cryostat sections have been regularly performed (see also previous sections).

### Light microscopic immunolocalization of striatin in cultured epithelial and myocardiac cells

Localization studies of striatin with α- and β-catenin, with other AJ markers and with desmosomal molecules have also been performed on cell culture monolayers, including epithelium or carcinoma-derived cells and cardiomyocyte-derived cells (for biochemical demonstrations of the presence of striatins in such cells, see Fig. 1a, b and Electronic supplementary material, Fig. S1; for immunofluorescence microscopy, see Electronic supplementary material, Figs. S7–S10). Striatins have been identified as major components not only in the cell-cell connecting *zonulae adhaerentes* of primary cultures. Figure S7, for example, shows a monolayer culture of rat myocardiac cells taken 2 days after birth (cf. Pieperhoff et al. 2012). This micrograph also demonstrates the absence of striatin in certain desmosome-related structures, including the variously sized intracellular assemblies of desmoplakin-rich material. Striatin has also been seen in continually-appearing cell-cell contact AJs of all epithelial cell culture monolayers examined (Figs. S8–10 present examples of the human breast carcinoma line MCF-7). Thus, striatins have to be counted among the obligatory constituents of AJs in tissues and in cell cultures. As striatins also occur in single cells in culture and in the living mammalian body (see Fig. 1 and Electronic supplementary material, Figs. S6–S9), one has to conclude that the synthesis and stability of striatin(s) are not dependent on established cell-cell junctions.

Remarkably, the integration of striatin(s) into the AJ *zonula* plaque structures is not restricted to completed assembly at the cell-cell contacts but can be detected in small *puncta*- or *fascia*-like structures in the cytoplasm, even in juxtanuclear regions, or in short plasma membrane intercepts before the formation of a continuous *zonula adhaerens* (Figs. S8, S9). In such situations, we find it especially surprising that even the newly formed AJ molecules are often closely associated with TJ proteins such as claudins and/or occludin (Figs. S8–S10).

### Immunoelectron microscopic localizations of striatins

**Fig. 9:**
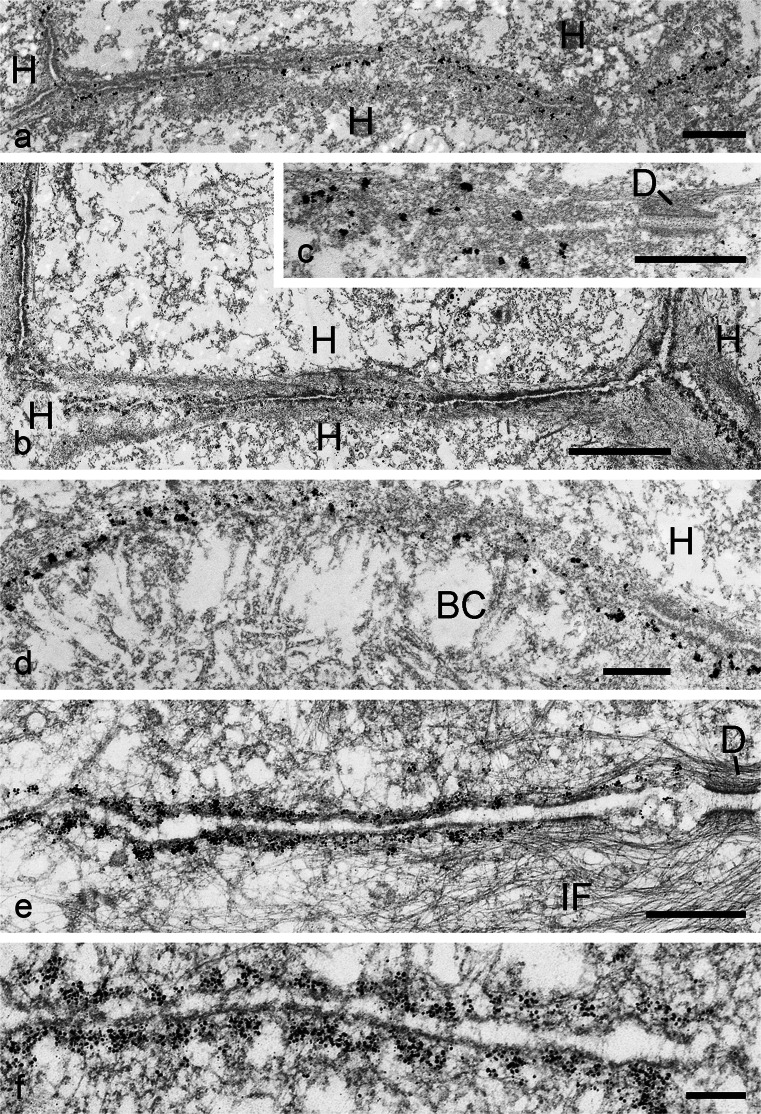
Immunoelectron microscopic localization of striatins in cytoplasmic plaques of adherens junctions in a simple epithelial tissue and an epithelium-derived cell culture. **a–c** Silver-enhanced immunogold reactions on ultracryotome sections of bovine liver tissue, showing that striatins are components of the adherens junction plaques in the interdesmosomal regions connecting here three (**a**) or four (**b**) hepatocytes (*H*), whereas desmosomes (*D* in **c**) are negative. **d** The specificity of adherens junctions is also evident from their localization in the entire subapical *zonula adhaerens* surrounding the bile canaliculi (*BC*). Note that, in the intracanalicular space, the villus-like apical cell processes are negative. **e**, **f** Silver-enhanced immunogold reaction of striatin antibodies in the entire adherens junction region between the desmosomes (*D*) in a monolayer cell culture of human breast-carcinoma-derived MCF-7 cells (*IF* bundles of intermediate-sized filaments). Note in **b** that the striatin label is exclusively seen in the submembranous plaque. *Bars* 2 μm (**b**), 1 μm (**a**), 500 nm (**c**-**e**), 200 nm (**f**)

Using snap-frozen tissues or monolayer cell cultures, we have been able to localize specific striatins on the plaques of cell-cell contact regions of the AJ type, as is shown for bovine liver tissue in Fig. 9a, b. Again, desmosomes or other categories of junctions (gap junctions, TJs) are not immunogold-labelled at all (e.g. the junction labelled D in Fig. 9c). Moreover, the immunogold reaction sites are all associated with cytoplasmic plaques of AJ strucures. Particularly eye-catching in this tissue is the *zonula adhaerens* labelling extending over the subapical junction region bordering on the bile canaliculi (BC in Fig. 9d), whereas no striatin label is associated with the apical villus-like cytoplasmic processes extending into the bile canalicular interior.

Even more extended and often dense immunolocalization reaction products have been seen in the plaques of interdesmosomal AJ-type cell-cell contact regions of densely grown monolayers of human breast carcinoma MCF-7 cells (Fig. 9e, f), hepatocellular carcinoma PLC and colon carcinoma CaCo2 cells (not shown) and in human epidermis-derived HaCaT tumour cells (not shown). Here, large amounts of antibody-bound heavy metal grains are exclusively concentrated on the submembranous AJ plaques (see Fig. 9f), whereas neither gap junctions nor TJs show marked enrichment.

**Fig. 10:**
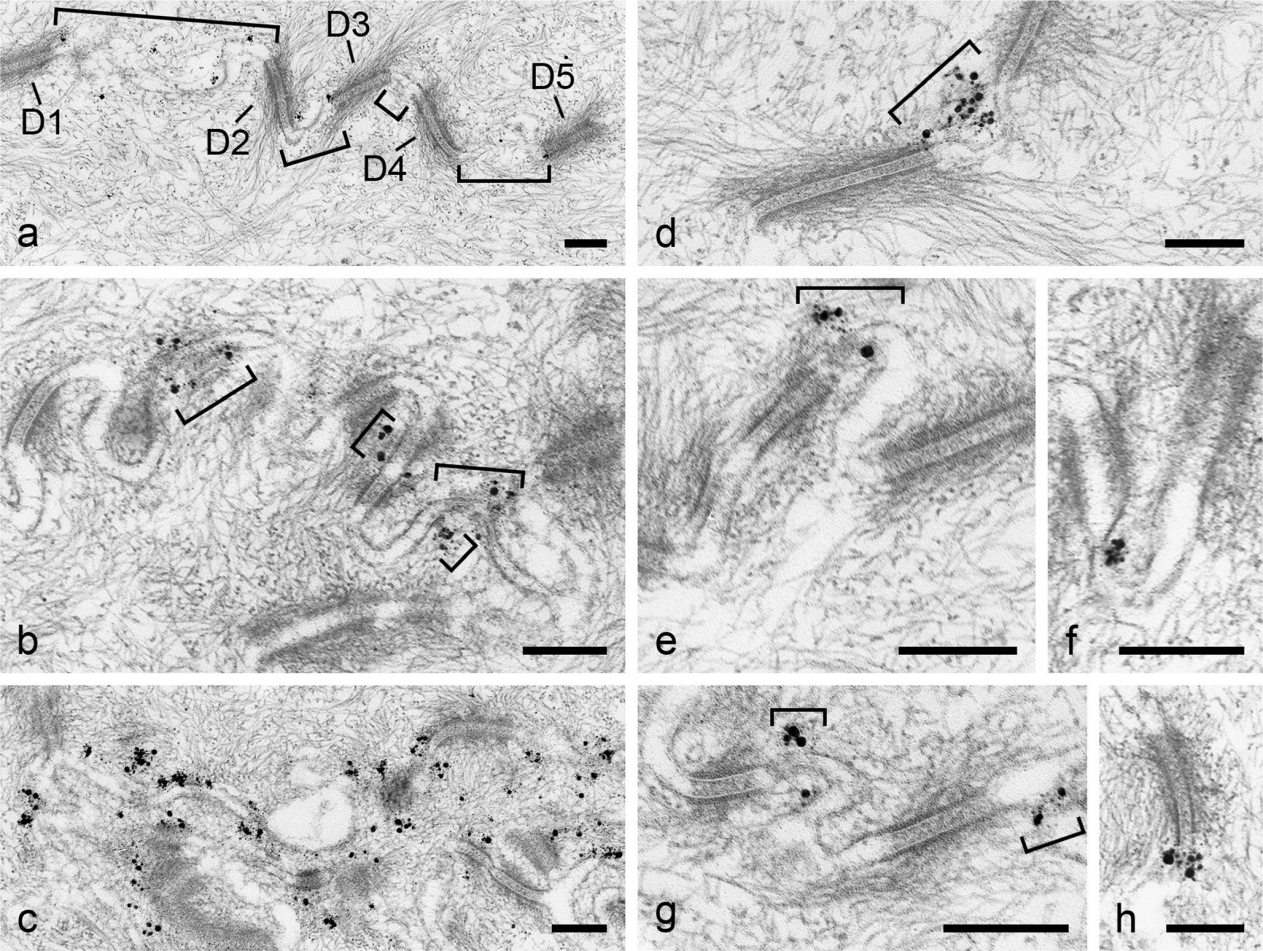
Immunoelectron microscopic localization of striatins in small *punctum*- or *fascia*-like adherens junctions or “molecular islands” in the interdesmosomal regions of the tessellate junctions of a stratified epithelium (here, bovine tongue mucosa). **a–c** Silver-enhanced immunogold reaction sites of antibodies against striatins on cryotome sections through bovine tongue mucosa fixed and embedded as described and further sectioned into ultrathin sections. The exposure times to striatin antibodies in the samples shown were 5, 7 and 9 min, respectively. **a** Survey micrograph showing the small original reaction sites (*dots* gold grains) in the interdesmosomal regions (*D1–D5* desmosomes, *brackets* tessellate junction regions). Note the abundance of bundles of intermediate-sized filaments (IFs) of the keratin type in the cytoplasm. **b** As in **a**, more than seven desmosomes occur in this region, with relatively high label densities (*brackets*) in variously sized and variously positioned interdesmosomal cell-cell contact regions. **c** Higher label densities showing the confinement of the striatin reaction sites to interdesmosomal regions. **d** Higher magnification showing an intensely immunogold-labelled junction cluster in an interdesmosomal tessellate junction region. **e** Higher magnification revealing a cluster of both small and larger heavy metal grains of a striatin reaction in a small *punctum adhaerens*-like junction island. **f–h** Striatin cluster (*bottom* in **f**) next to the edge of a desmosome (**f**), small interdesmosomal islands of immunolabelled *puncta adhaerentia* (*brackets*), and a striatin-rich cell-cell contact island (*bottom* in **h**) in direct association with the margin of a desmosome. *Bars* 200 nm

In stratified epithelia, immunoelectron reaction is also restricted to interdesmosomal regions. Figure 10a-h shows striatin labelling by gold-silver grains at such regions (desmosomes are numbered in Fig. 10a) with only a few small immunogold grains. By contrast, Fig. 10b presents clusters of larger metal label grains in positions that, in some cases, might be equivalent to local *puncta adhaerentia* within a tessellate junction (regions denoted by brackets in Fig. 10b). An even more extended and intensive interdesmosomal striatin labelling pattern is seen in Fig. 10c). In general, the sizes and the relative positions of striatin immunoreaction products vary (for light microscopic comparisons, see Electronic supplementary material, Fig. S5). In Fig. 10d, the label extends over the entire interdesmosomal region, whereas the antibody-linked heavy metal grains in Fig. 10e-h show a higher tendency to cluster in regions near desmosome margins. In view of the frequent close vicinity of striatin localization sites with desmosomal margins (see above), we have examined the proteins of desmosomal fractions from calf muzzle epidermis, tongue mucosa and oesophagus tissue prepared in the Heidelberg laboratory (see Mueller and Franke 1983; Kapprell et al. 1985) by SDS-PAGE and immunoblotting, with and without immunoprecipitation and protein cross-linking experiments (cf. Straub et al. 2011; Pieperhoff et al. 2012) but we have found no indications of the occurrence of striatins within desmosomal structures.

## Discussion

Members of the striatin family of proteins are by no means specific for, or especially abundant in, neuronal or other cells of the nervous system as originally reported (for references see Introduction). As shown in this study, they are synthesized and integrated into protein complexes, dispersed particles or plaques of cell junctions of most, if not all mammalian cells, in single cells and in cultured cells and cells of tissues (see also Hwang and Pallas 2013).

For the sake of clarity, we consider it important to emphasize that our results show that the classification of members of the striatin family as “desmosomal proteins” (Meurs et al. 2013) is not correct. We have not detected striatin(s) in any type of desmosome or desmosome-related structure. On the contrary, striatins are exclusive and constitutive components of AJs such as the *zonulae adhaerentes* of polar epithelia, of certain punctate or fascia-like substructures of the tessellate junctions in stratified epithelia, and of the CJs in the intercalated disks of mammalian heart (for the latter localization in boxer dog, see also Meurs et al. 2010). Consequently, striatins (certainly the major cardiac isoform) should be added to the diagnostic “control list” of markers for hereditary AC and DC damage (Table 2; cf. Asimaki et al. 2009).

**Table 2:** Constitutive molecules of composite junctions (*areae compositae*) in the intercalated disks of mammalian cardiomyocytes

**Transmembrane cadherins**	**Plaque proteins**
**Adherens junction molecules**	**Adherens junction molecules**
N-cadherin	α-Catenin (αE + αT)
Cadherin-11	β-Catenin
	Protein p120
	Protein p0071
	Protein ARVCF
	Plakoglobin
	Myozap
	Striatin(s)
	Protein ZO-1
**Desmosomal molecules**	**Desmosomal plaque molecules**
Desmoglein 2 (Dsg2)	Plakoglobin
Desmocollin 2 (Dsc2)	Plakophilin 2 (Pkp2)
	Desmoplakin
**Other molecules**	
Plectin	
Ankyrin-G	
Protein PERP	
Protein LUMA	

*Armadillo* repeats-containing proteins are underlined

Our results also clearly show that striatin proteins are not components of TJs, although they can often be seen in the vicinity of TJs. The most convincing argument for this conclusion is presented by the CJs of the myocardial intercalated disks from which TJ-specific molecules and structures are totally absent. Similarly convincing examples are provided by several cultured cells with large regions positive for striatins and other AJ molecules but negative for TJ markers.

Additional locations of members of this protein family might occur in which the striatins as scaffold proteins are masked by specific complex partner molecules. Indeed, the members of this protein family are typical scaffold proteins able to form not only oligomeric complexes, but also complex “multimodular” structures, including some involved in diverse signalling and regulatory functions (see Muro et al. 1995; Castets et al. 1996, 2000; Bartoli et al. 1998, 1999; Kachidian et al. 1998; Salin et al. 1998; Moreno et al. 2000, 2001; Baillat et al. 2001; Gaillard et al. 2001; Yu et al. 2001; Lu et al. 2004; Joshi-Mukherjee et al. 2008; Gordon et al. 2011; Chen et al. 2012; for a recent general review on scaffold proteins see Garbett and Bretscher 2014). Consequently, we now need to examine the possible occurrence of such striatin-binding proteins and striatin-typical functions in plaque complexes of, for example, epithelial AJs and myocardiac CJs. Similarly important are detailed experimental analyses, including gene knock-out studies, to determine which of the so highly related and so similarly sized striatin forms are involved in certain pathogenic conditions, such as the aforementioned cardiac AC and DC damage.

Whether the pathogenic effects of certain CJ molecule mutations on cardiomyopathies such as ACs or Brugada syndrome take place in junctions between cardiomyocytes or in any of the special junctions connecting the conductive Purkinje fibre cells (for details, see Pieperhoff et al. 2010; Mezzano et al. 2014) remains unclear. Also unknown is whether these effects are direct or indirect, e.g. by involving connexin43 or connexin40 of the intimately associated gap junctions or the Nav1.5 sodium ion channels, and decisive experimental results are needed. Such indirect reactions via other adjacent cell-cell contact structures have repeatedly been discussed by a series of authors (Oxford et al. 2007a, b, 2011; Sato et al. 2009, 2011; Cerrone et al. 2012, 2014; Delmar and Liang 2012; Gomes et al. 2012; Agullo-Pascual et al. 2013, 2014; Meens et al. 2013; Noorman et al. 2013; Cerrone and Delmar 2014; Lyon et al. 2014; Vreeker et al. 2014; for earlier references and general reviews, see also Saffitz 2007, 2011; Delmar and McKenna 2010; Delmar and Makita 2012; Murray 2012; Rickelt and Pieperhoff 2012; Rizzo et al. 2012a, b). Finally, we cannot yet exclude that both direct and indirect effects of the mutated or otherwise modified molecules contribute to the pathogenetic effects mentioned. One hope is that detailed studies of the animal pathogenesis examples and transgenic experimental possibilities (for some related references, see Table 3 in Rickelt and Pieperhoff 2012; see also Fox et al. 2007; Vatta et al. 2007) will help in elucidating the cardiomyopathic mechanisms involved.

The recognition of striatin as a multimodular scaffolding protein that occurs in the cytoplasmic plaques of various kinds of cell junctions has also to be discussed in comparison with other scaffolding plaque proteins such as protein ZO-1 of various tight and adherens junctions (AJs), as well as AJ α-catenin and the ERM protein-binding component EBP50 found at the actin microfilament associations with microvillar membranes (for a review, see Garbett and Bretscher 2014). Such scaffolding proteins can also form a diversity of other complexes located in other structures or regions in which they might be masked for certain cell-type-specific antibodies but do indeed bind to antibodies reactive with other epitopes on the same molecule. As a result, the same scaffolding protein might immunocytochemically appear positive in one structure but negative in another. Consequently, such selective masking of one conformational domain might result in completely negative immunolocalization sites of the same protein that is positive with other antibodies (see, for example, the results obtained for protein LUMA in the report of Franke et al. 2014).

## Electronic supplementary material

Below is the link to the electronic supplementary material.

Fig. S1 Characterizations of various antibodies to striatins by immunoblot reactions with SDS-PAGE-separated polypeptides. The lanes contain total protein lysates from cultures of human MCF-7 mammary carcinoma cells (*lane* 1), of human glioma cells (line U333/MG, *lane* 2), and from homogenates of adult human heart tissue (*lane* 3). The specific striatin antibodies used were the mAb against striatin(s) of Becton-Dickinson (**a**) and guinea pig antibodies NTB (**b**), 268B (**c**) and 301B (**d**). Note that the antibodies used in **a** reveal two equally reactive bands of ca. 113 and 98 kDa whereas the guinea pig antibodies used react only with the higher M_r_ band. Note further that the M_r_ band of 113 kDa in *lane* 3 shows a relatively intense reaction in **a** and **c** but only weak reactions in **b** and **d** (*lane* 3 of **b** and **d**, shown after prolonged exposure) (GIF 27 kb)

High Resolution (TIFF 423 kb)

Fig. S2 Double-label, confocal laser-scanning immunofluorescence microscopy of cryostat sections through bovine myocardium, showing the differential effects of repeated washings on the immunolocalization of some specific proteins. **a**, **a''**, **a'''** Immunostaining with mouse antibodies against desmoplakin (**a**, *red*; mAb m) compared with that obtained with guinea pig antibodies against plectin (**a'**-**a'''**, *green*) as seen after limited washings (**a'**-**a'''**; for details see Materials and methods). Note that the composite junctions (CJs) of the intercalated disks (IDs) are intensely labelled but that the sarcomeric Z-bands are also somewhat stained. **b**–**b'''** Immunostaining as in **a**–**a'''** but with additional intensive buffer washes (here an additional 5-min wash with PBS containing 0.2% Triton X-100) in order to remove the stickily adsorbed plectin antibodies from the sarcomeric Z-bands. Note that in all immunostaining images there is no residual plectin staining on Z-bands. Figs. **a'''** and **b'''** are the same micrographs as **a''** and **b''** but with a phase contrast background. *Bars* 20 μm (GIF 226 kb)

High Resolution (TIFF 2713 kb)

Fig. S3 Double-label, confocal laser-scanning immunofluorescence microscopy of cryostat sections through boar myocardium, demonstrating the specificity of a sarcomeric component, α-actinin, for sarcomeric Z-bands and of striatin for the composite junctions in the intercalated disks. Striatin (*green*; pAb gp) is seen only on CJs, whereas α-actinin is seen only on Z-bands. *Bar* 10 μm (GIF 148 kb)

High Resolution (TIFF 1203 kb)

Fig. S4 Double-label, confocal laser-scanning immunofluorescence microscopy of boar myocardium, using two different types of α-actinin antibodies, a murine mAb, EA53, labelled in *red*, and rabbit pAb (both from Sigma) labelled in *green*. Note complete colocalization (*yellow* merger colour) and reactions on Z-lines only. *Bar* 20 μm (GIF 191 kb)

High Resolution (TIFF 2089 kb)

Fig. S5 Double-label, confocal laser-scanning immunofluorescence microscopy of a cryostat section through adult rat myocardium (fixation: 10 min acetone, washes, 5 min PBS containing 0.2% Triton X-100). Note the far-reaching colocalization of β-catenin (**a**, **a''**, **a'''**, *red*; mAb m) and striatin (**a'**-**a'''**, *green*; p301B gp) in composite junctions of the intercalated disks (**a'''** with a phase contrast background). *Bar* 20 μm (GIF 74 kb)

High Resolution (TIFF 807 kb)

Fig. S6 Double-label, confocal laser-scanning immunofluorescence microscopy showing the localization of ankyrin-G as a component of the CJs in cryostat sections through various mammalian myocardia. **a**-**a'''** Micrographs showing a human myocardium immunostained for ankyring-G in comparison with desmoplakin as a demonstration of the far-reaching colocalization of desmoplakin (**a**, *red*; mAb m) and ankyrin-G (**a'**, *green*; pAb goat), shown in both colour channels (**a''**, *yellow* merge colour) without and with phase contrast background (**a'''**). The inserts demonstrate that colocalization of these two CJ plaque proteins is not only seen in well developed intercalated disks but also in small, even tiny CJ structures (see *yellow* whiskers and dots in the insert). **b**–**b'''** Micrographs showing boar myocardium immunostained in parallel to the human sample in **a**-**a'''** (same details). **c**, **c'** Micrographs showing the CJ co-localization of desmoplakin (**c'**, *green*) and ankyrin-G (**c**, *red*) in bovine myocardium (**c'**, *yellow* merger colour). **d**-**d'''** Micrographs showing the immunolocalization of ankyrin-G (**a**-**d'''**, *red*) and desmoplakin (**d**, *green*) or striatin (**f**) in CJ structures of rat myocardium on a phase contrast background (*yellow* merger colour). *Bars* 20 μm and 10 μm (*insets* in **a** and **b**) (GIF 413 kb)

High Resolution (TIFF 4866 kb)

Fig. S7 Double-label, confocal laser-scanning immunofluorescence microscopy of a dense-grown monolayer culture of primary cultured cells from perinatal rat hearts, originally taken at day 2 after birth. The cells still show positivity for striatin (**a,** *red*; mAb m) and desmoplakin (**a'**, *green*; pAb gp) both of which, however, are now located only partially at cell-cell junction sites, here and there also in colocalization (**a''**, *yellow* merger colour), whereas others are revealed as separate structures of either *red* or *green* colour located on plasma membranes or dissociated into the cytoplasm where variously-sized, *green*-coloured aggregates, i.e. desmoplakin structures, predominate. *Bar* 20 μm (GIF 150 kb)

High Resolution (TIFF 1369 kb)

Fig. S8 Double-label, confocal laser-scanning immunofluorescence microscopy of a monolayer culture of human mammary carcinoma-derived cells of line MCF-7, showing extensive colocalization of striatin (**a**, *red*; mAb m) and β-catenin (**a'**, *green*; rb Ab) at cell-cell contacts, locally often resulting in the appearance of *yellow* merger colour (**a''**, on a phase contrast background). *Bar* 20 μm (GIF 180 kb)

High Resolution (TIFF 2492 kb)

Fig. S9 Double-label, confocal laser-scanning immunofluorescence microscopy showing monolayer culture MCF-7 cells (2 days after trypsin-dissociation and re-seeding), double-stained with antibodies against striatin (**a**, *red*; mAb m) and rabbit antibodies against occludin (**a'**, *green*; rb Ab), in **a''** on the background of the phase contrast picture. Note that the process of reformation of cell-cell junctions, i.e. of adherens junctions and tight junctions, is not yet complete (e.g., in the upper portion of the picture) but that here both processes, the reformations of a tight junction *zonula occludens* (occludin) and that of a *zonula adhaerens* (striatin), are coordinated in time and space, as indicated by the extensive colocalization (*yellow* merger colour) of both marker proteins in extended junctional structures and in small, isolated intercepts which appear as individual dots or *fasciae* on plasma membranes or elsewhere in the cytoplasm (e.g. in the upper left region). *Bar* 20 μm (GIF 96 kb)

High Resolution (TIFF 1050 kb)

Fig. S10 Micrograph showing another region of the MCF-7 cell culture immunostaining preparation presented in the preceeding figure. Note here that during the reformation of the cell-cell contact regions of associated tight and adherens junctions (*zonulae occludentes* and *zonulae adhaerentes*) extended cell-cell contact regions with merger colour (*yellow*) are seen as well as smaller, sometimes tiny structures which are characterized by *yellow* merger colour, positive for markers of both tight and adherens junctions of widely different lengths. These can occur not only near plasma membranes but also deep in the cytoplasm, partly even in the perinuclear region (e.g., in the upper marginal part of the picture). *Bar* 20 μm (GIF 126 kb)

High Resolution (TIFF 1537 kb)

Table S1 Primary antibodies (DOC 79 kb)
