# Supplementary figures and images for: Striatins as plaque molecules of zonulae adhaerentes in simple epithelia, of tessellate junctions in stratified epithelia, of cardiac composite junctions and of various size classes of lateral adherens junctions in cultures of epithelia- and carcinoma-derived cells
Source: Cell Tissue Res. 2014 Dec 12;359(3):779–97. doi: 10.1007/s00441-014-2053-z (PMC4341017; doi:10.1007/s00441-014-2053-z)

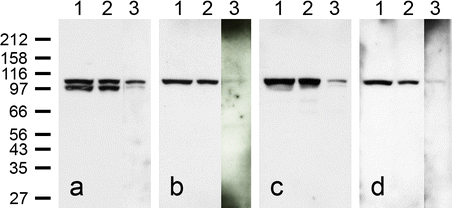

Supplement: Supplementary file 1 — Characterizations of various antibodies to striatins by immunoblot reactions with SDS-PAGE-separated polypeptides. The lanes contain total protein lysates from cultures of human MCF-7 mammary carcinoma cells (lane 1), of human glioma cells (line U333/MG, lane 2), and from homogenates of adult human heart tissue (lane 3). The specific striatin antibodies used were the mAb against striatin(s) of Becton-Dickinson (a) and guinea pig antibodies NTB (b), 268B (c) and 301B (d). Note that the antibodies used in a reveal two equally reactive bands of ca. 113 and 98 kDa whereas the guinea pig antibodies used react only with the higher Mr band. Note further that the Mr band of 113 kDa in lane 3 shows a relatively intense reaction in a and c but only weak reactions in b and d (lane 3 of b and d, shown after prolonged exposure) (GIF 27 kb) [file 441_2014_2053_Fig11_ESM.gif]

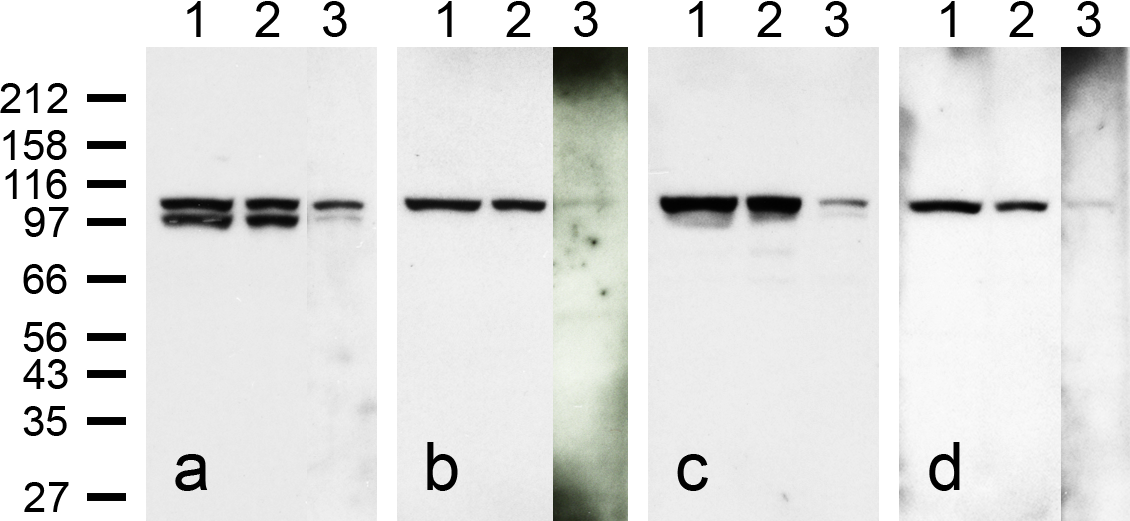

Supplement: Supplementary file 2 — High Resolution (TIFF 423 kb) [file 441_2014_2053_MOESM1_ESM.tif]

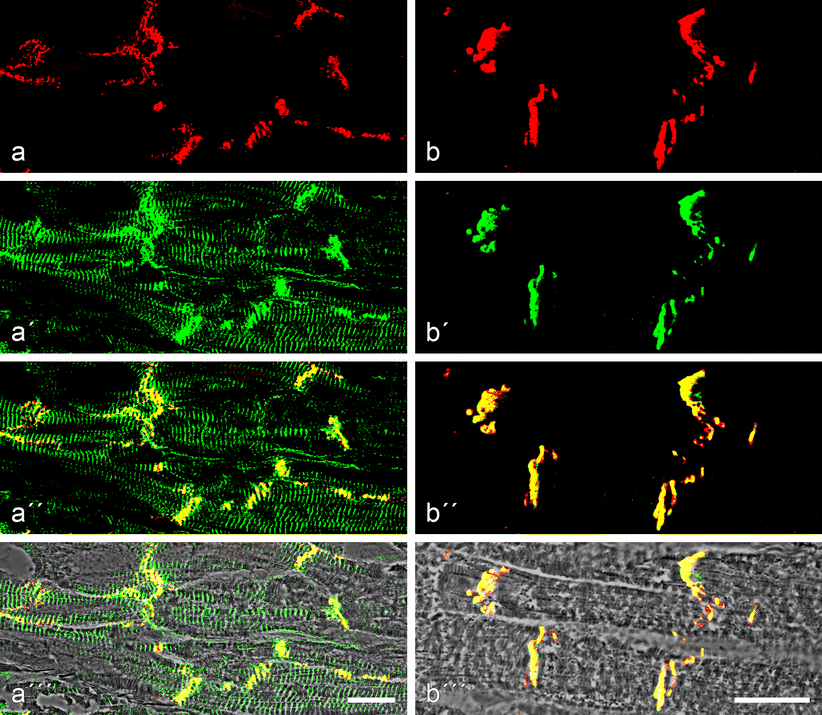

Supplement: Supplementary file 3 — Double-label, confocal laser-scanning immunofluorescence microscopy of cryostat sections through bovine myocardium, showing the differential effects of repeated washings on the immunolocalization of some specific proteins. a, a'', a''' Immunostaining with mouse antibodies against desmoplakin (a, red; mAb m) compared with that obtained with guinea pig antibodies against plectin (a'-a''', green) as seen after limited washings (a'-a'''; for details see Materials and methods). Note that the composite junctions (CJs) of the intercalated disks (IDs) are intensely labelled but that the sarcomeric Z-bands are also somewhat stained. b–b''' Immunostaining as in a–a''' but with additional intensive buffer washes (here an additional 5-min wash with PBS containing 0.2% Triton X-100) in order to remove the stickily adsorbed plectin antibodies from the sarcomeric Z-bands. Note that in all immunostaining images there is no residual plectin staining on Z-bands. Figs. a''' and b''' are the same micrographs as a'' and b'' but with a phase contrast background. Bars 20 μm (GIF 226 kb) [file 441_2014_2053_Fig12_ESM.gif]

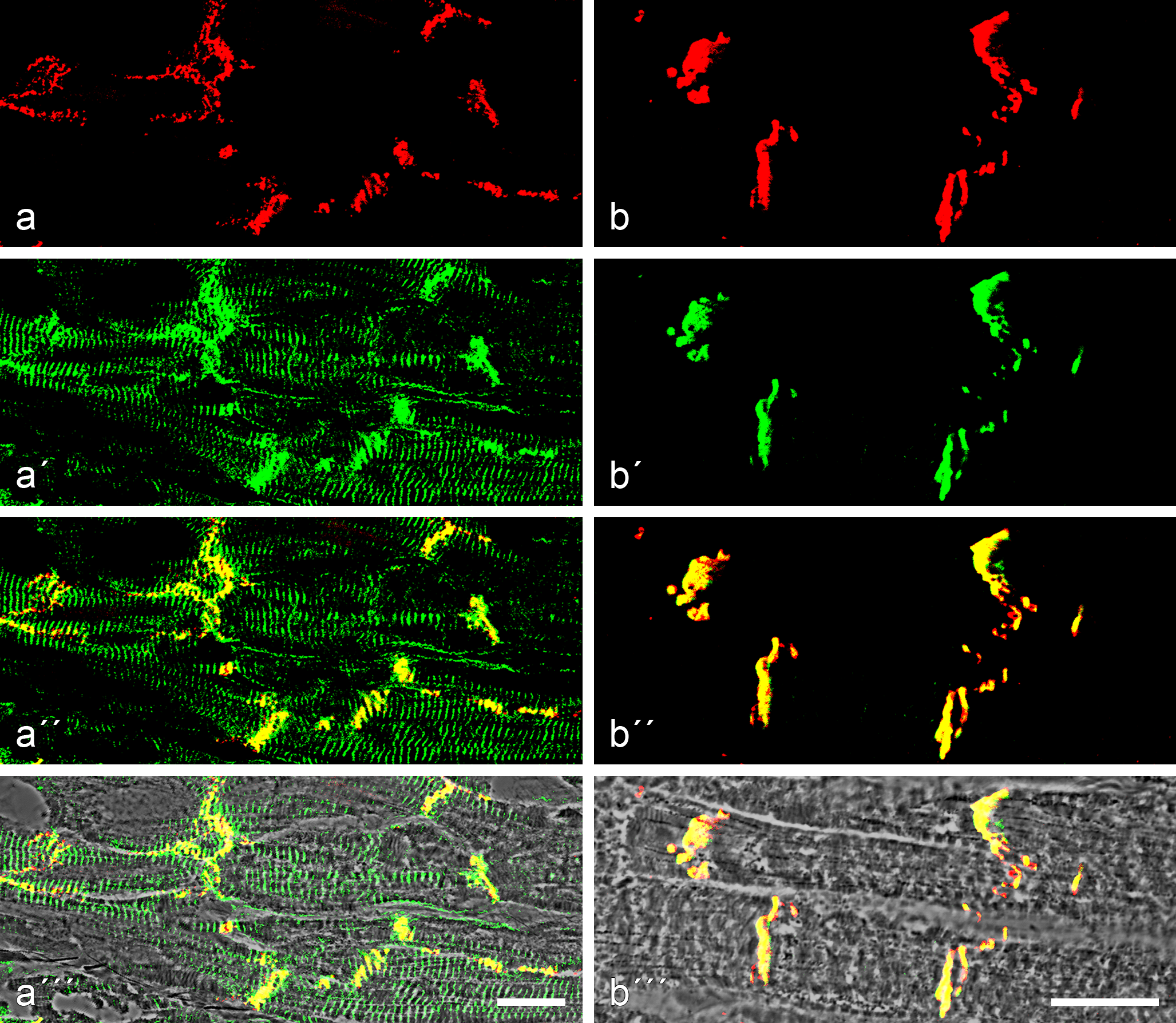

Supplement: Supplementary file 4 — High Resolution (TIFF 2713 kb) [file 441_2014_2053_MOESM2_ESM.tif]

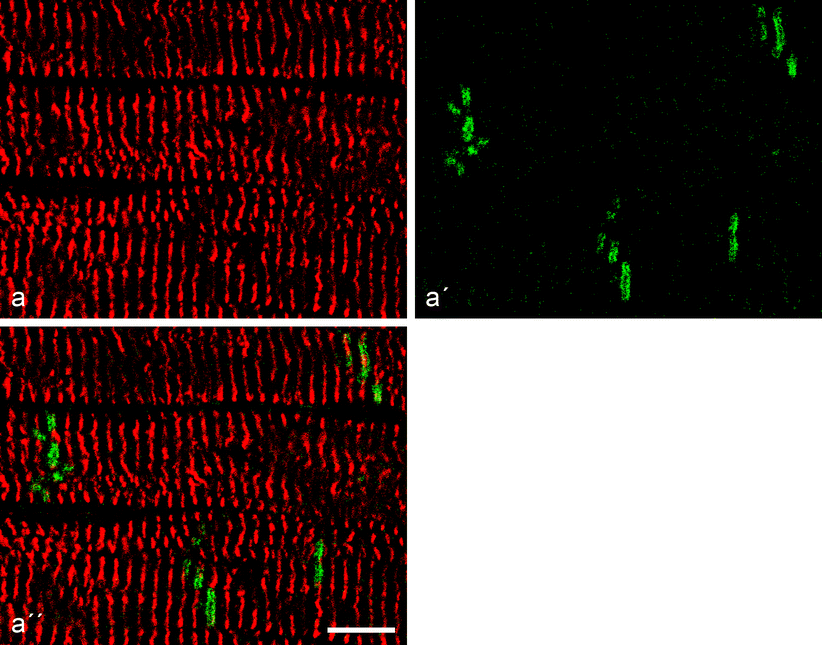

Supplement: Supplementary file 5 — Double-label, confocal laser-scanning immunofluorescence microscopy of cryostat sections through boar myocardium, demonstrating the specificity of a sarcomeric component, α-actinin, for sarcomeric Z-bands and of striatin for the composite junctions in the intercalated disks. Striatin (green; pAb gp) is seen only on CJs, whereas α-actinin is seen only on Z-bands. Bar 10 μm (GIF 148 kb) [file 441_2014_2053_Fig13_ESM.gif]

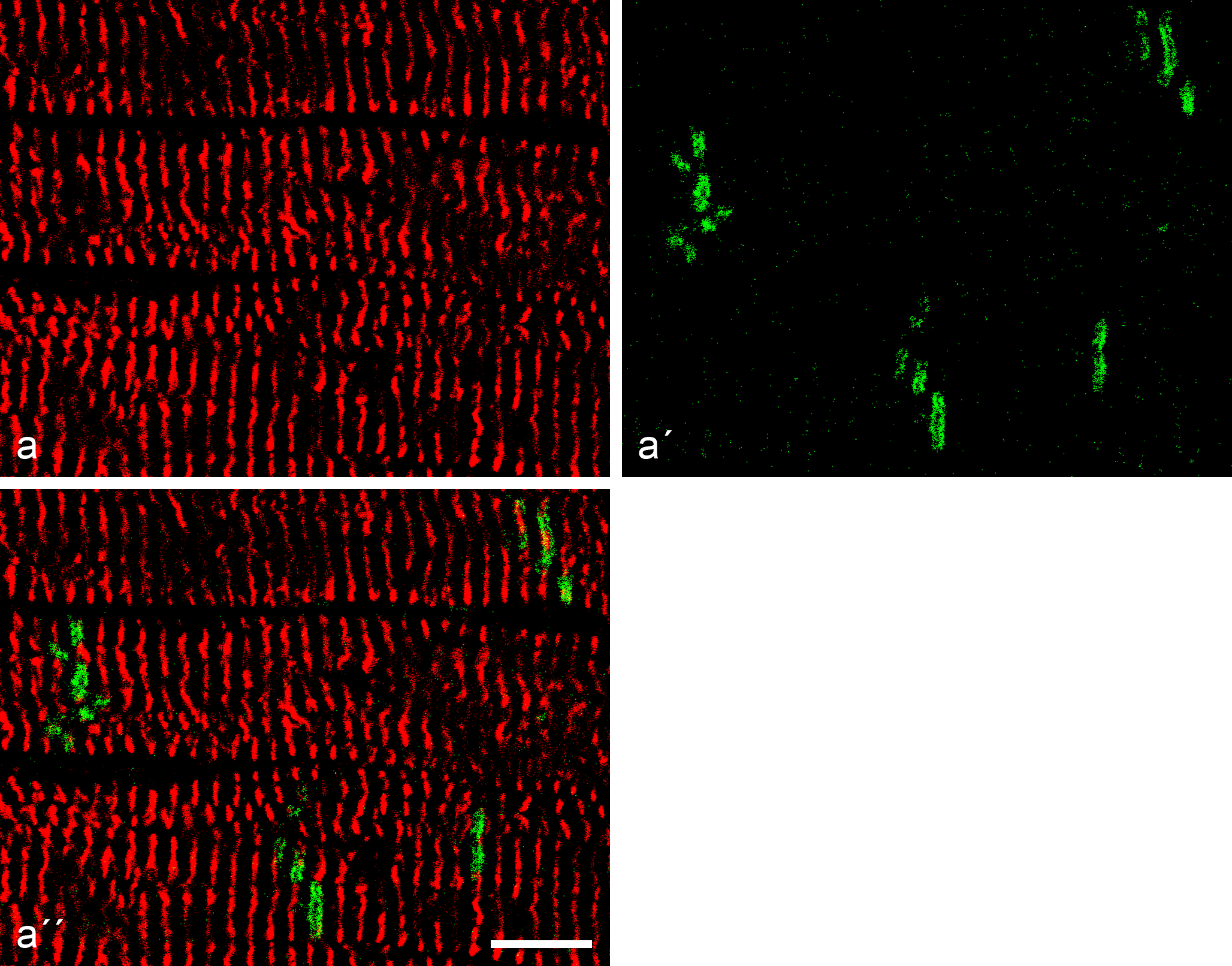

Supplement: Supplementary file 6 — High Resolution (TIFF 1203 kb) [file 441_2014_2053_MOESM3_ESM.tif]

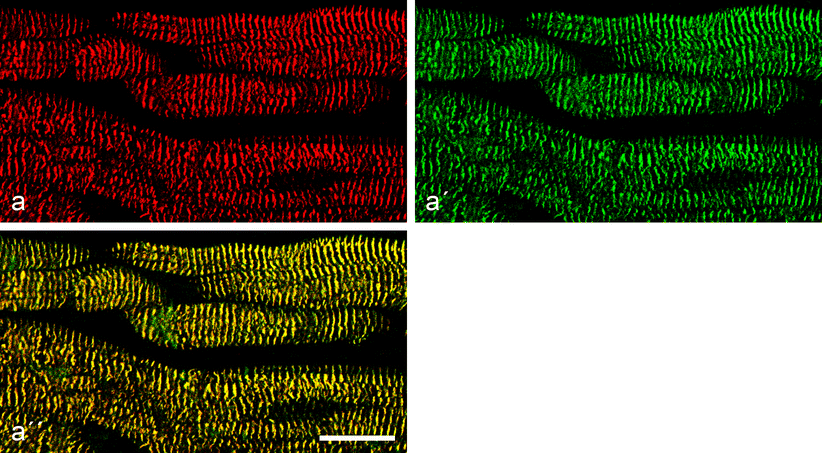

Supplement: Supplementary file 7 — Double-label, confocal laser-scanning immunofluorescence microscopy of boar myocardium, using two different types of α-actinin antibodies, a murine mAb, EA53, labelled in red, and rabbit pAb (both from Sigma) labelled in green. Note complete colocalization (yellow merger colour) and reactions on Z-lines only. Bar 20 μm (GIF 191 kb) [file 441_2014_2053_Fig14_ESM.gif]

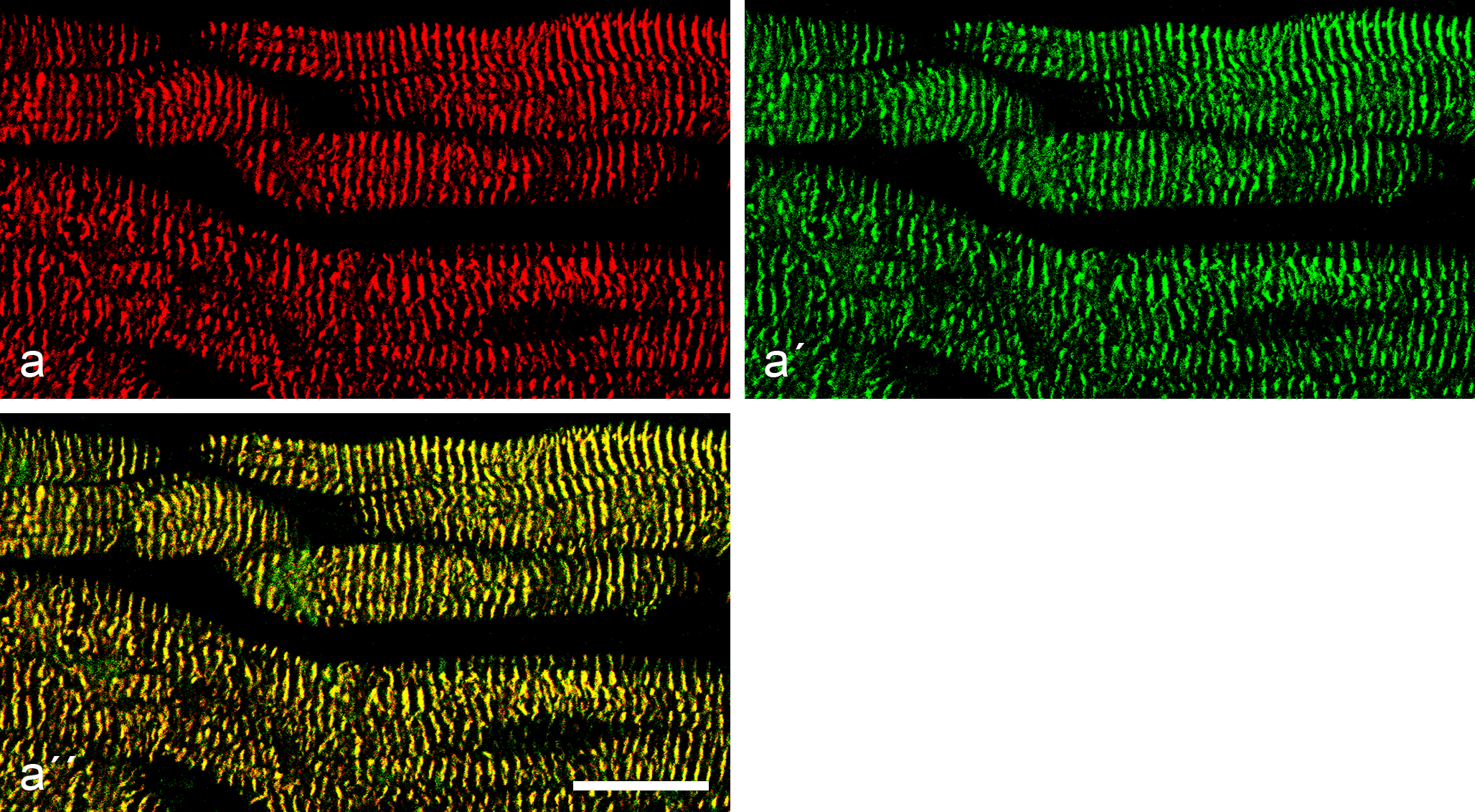

Supplement: Supplementary file 8 — High Resolution (TIFF 2089 kb) [file 441_2014_2053_MOESM4_ESM.tif]

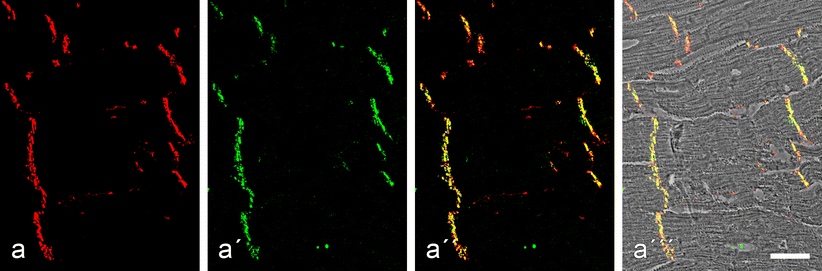

Supplement: Supplementary file 9 — Double-label, confocal laser-scanning immunofluorescence microscopy of a cryostat section through adult rat myocardium (fixation: 10 min acetone, washes, 5 min PBS containing 0.2% Triton X-100). Note the far-reaching colocalization of β-catenin (a, a'', a''', red; mAb m) and striatin (a'-a''', green; p301B gp) in composite junctions of the intercalated disks (a''' with a phase contrast background). Bar 20 μm (GIF 74 kb) [file 441_2014_2053_Fig15_ESM.gif]

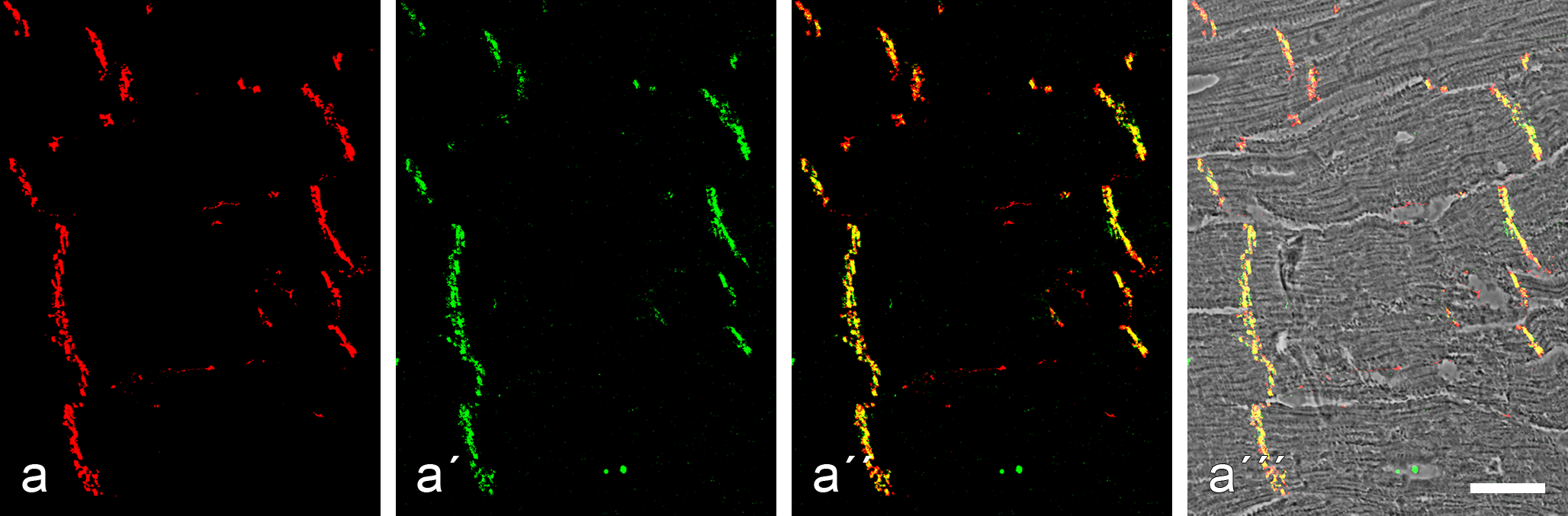

Supplement: Supplementary file 10 — High Resolution (TIFF 807 kb) [file 441_2014_2053_MOESM5_ESM.tif]

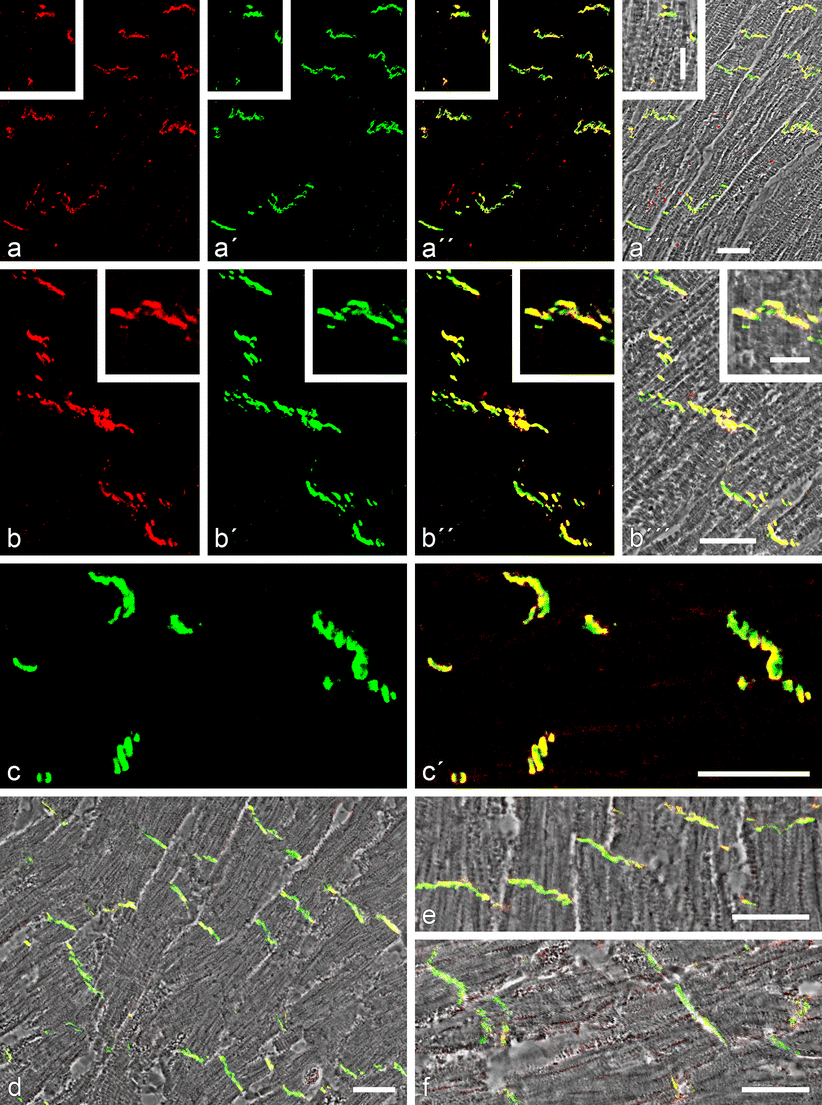

Supplement: Supplementary file 11 — Double-label, confocal laser-scanning immunofluorescence microscopy showing the localization of ankyrin-G as a component of the CJs in cryostat sections through various mammalian myocardia. a-a''' Micrographs showing a human myocardium immunostained for ankyring-G in comparison with desmoplakin as a demonstration of the far-reaching colocalization of desmoplakin (a, red; mAb m) and ankyrin-G (a', green; pAb goat), shown in both colour channels (a'', yellow merge colour) without and with phase contrast background (a'''). The inserts demonstrate that colocalization of these two CJ plaque proteins is not only seen in well developed intercalated disks but also in small, even tiny CJ structures (see yellow whiskers and dots in the insert). b–b''' Micrographs showing boar myocardium immunostained in parallel to the human sample in a-a''' (same details). c, c' Micrographs showing the CJ co-localization of desmoplakin (c', green) and ankyrin-G (c, red) in bovine myocardium (c', yellow merger colour). d-d''' Micrographs showing the immunolocalization of ankyrin-G (a-d''', red) and desmoplakin (d, green) or striatin (f) in CJ structures of rat myocardium on a phase contrast background (yellow merger colour). Bars 20 μm and 10 μm (insets in a and b) (GIF 413 kb) [file 441_2014_2053_Fig16_ESM.gif]

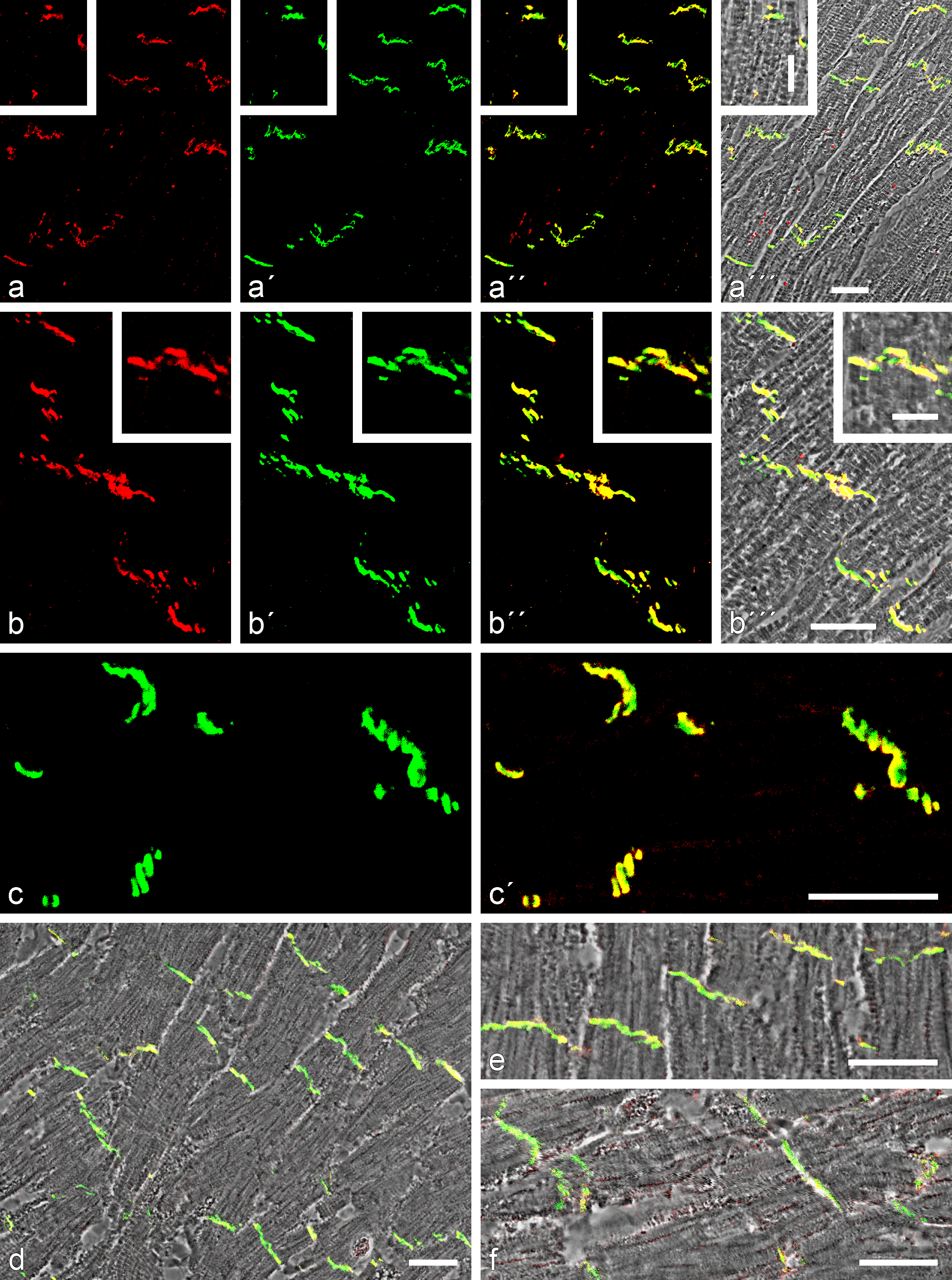

Supplement: Supplementary file 12 — High Resolution (TIFF 4866 kb) [file 441_2014_2053_MOESM6_ESM.tif]

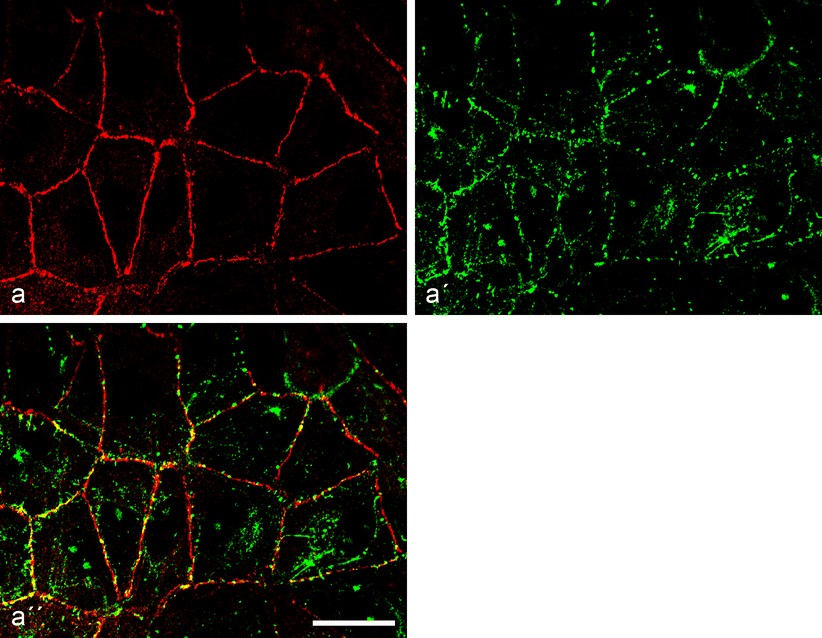

Supplement: Supplementary file 13 — Double-label, confocal laser-scanning immunofluorescence microscopy of a dense-grown monolayer culture of primary cultured cells from perinatal rat hearts, originally taken at day 2 after birth. The cells still show positivity for striatin (a, red; mAb m) and desmoplakin (a', green; pAb gp) both of which, however, are now located only partially at cell-cell junction sites, here and there also in colocalization (a'', yellow merger colour), whereas others are revealed as separate structures of either red or green colour located on plasma membranes or dissociated into the cytoplasm where variously-sized, green-coloured aggregates, i.e. desmoplakin structures, predominate. Bar 20 μm (GIF 150 kb) [file 441_2014_2053_Fig17_ESM.gif]

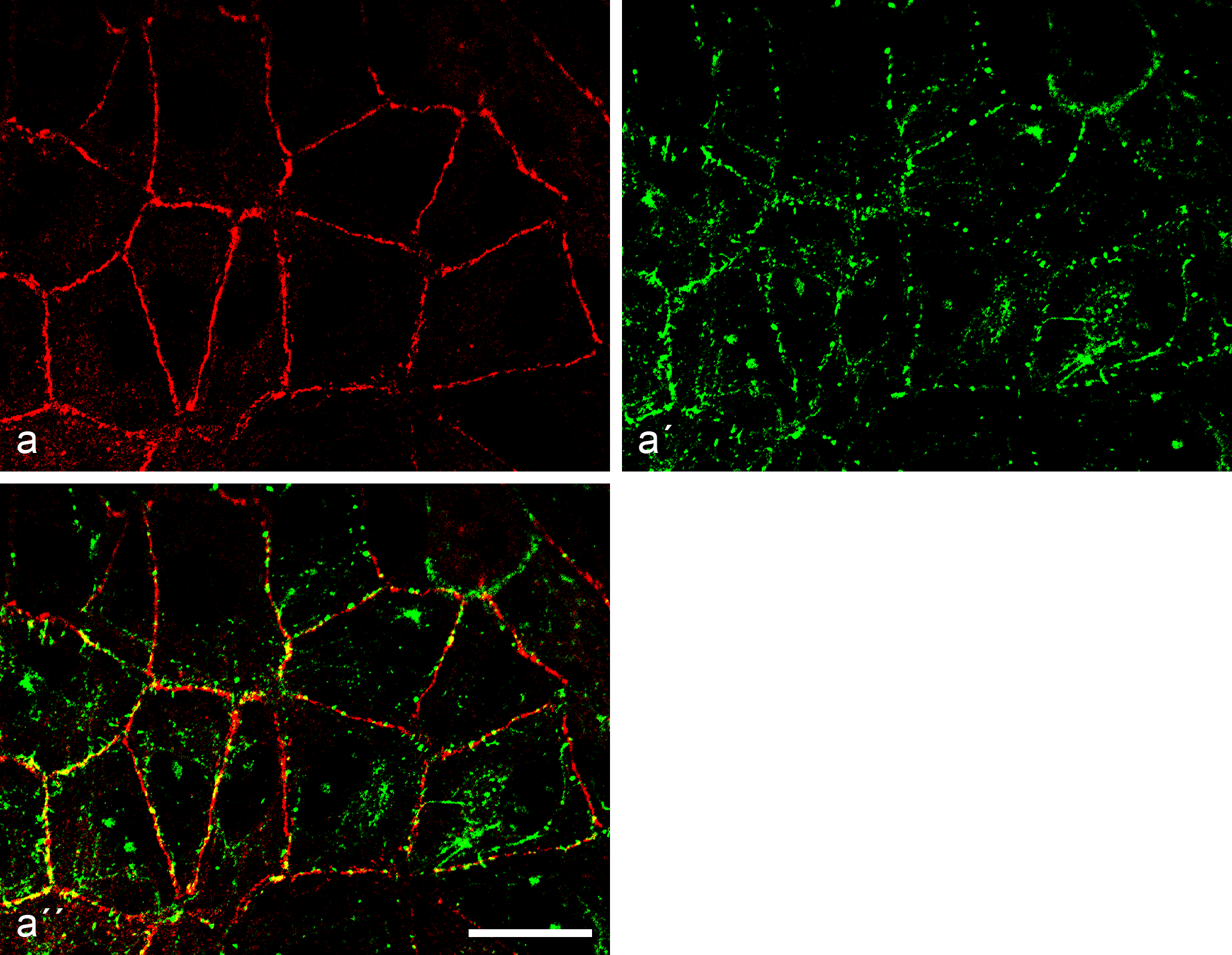

Supplement: Supplementary file 14 — High Resolution (TIFF 1369 kb) [file 441_2014_2053_MOESM7_ESM.tif]

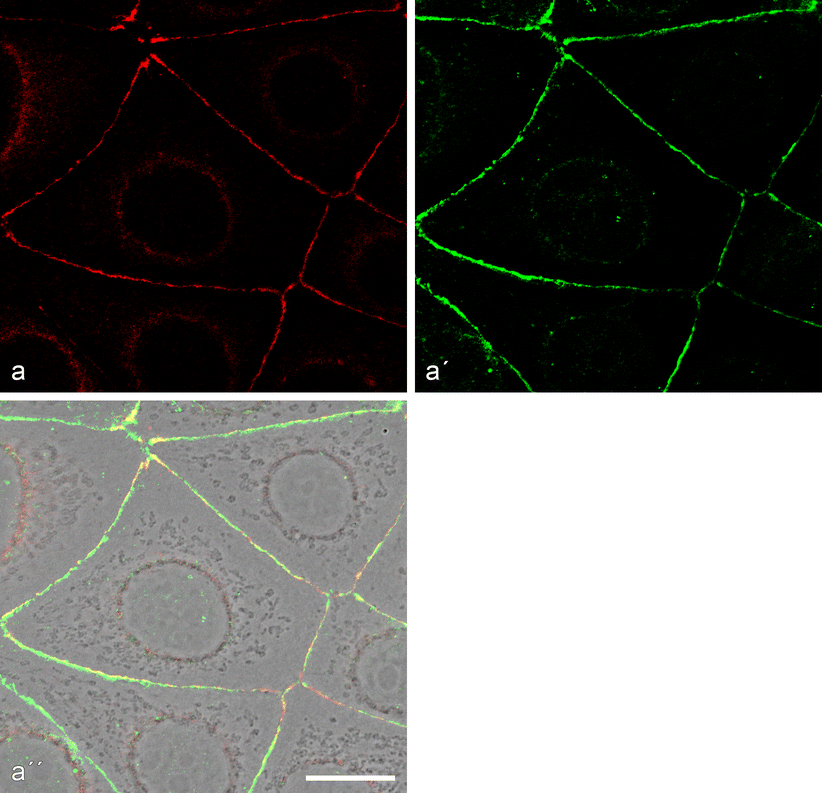

Supplement: Supplementary file 15 — Double-label, confocal laser-scanning immunofluorescence microscopy of a monolayer culture of human mammary carcinoma-derived cells of line MCF-7, showing extensive colocalization of striatin (a, red; mAb m) and β-catenin (a', green; rb Ab) at cell-cell contacts, locally often resulting in the appearance of yellow merger colour (a'', on a phase contrast background). Bar 20 μm (GIF 180 kb) [file 441_2014_2053_Fig18_ESM.gif]

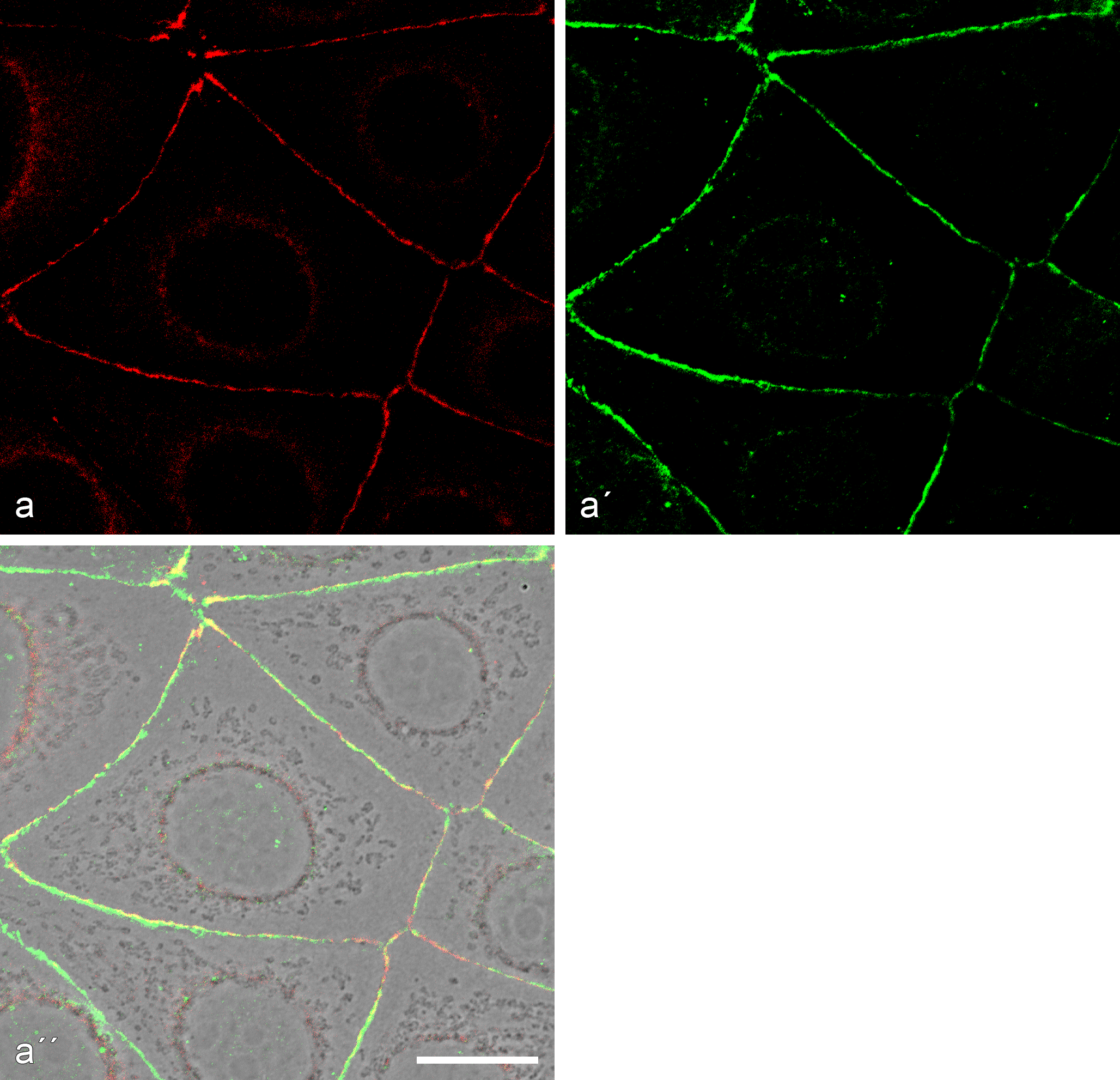

Supplement: Supplementary file 16 — High Resolution (TIFF 2492 kb) [file 441_2014_2053_MOESM8_ESM.tif]

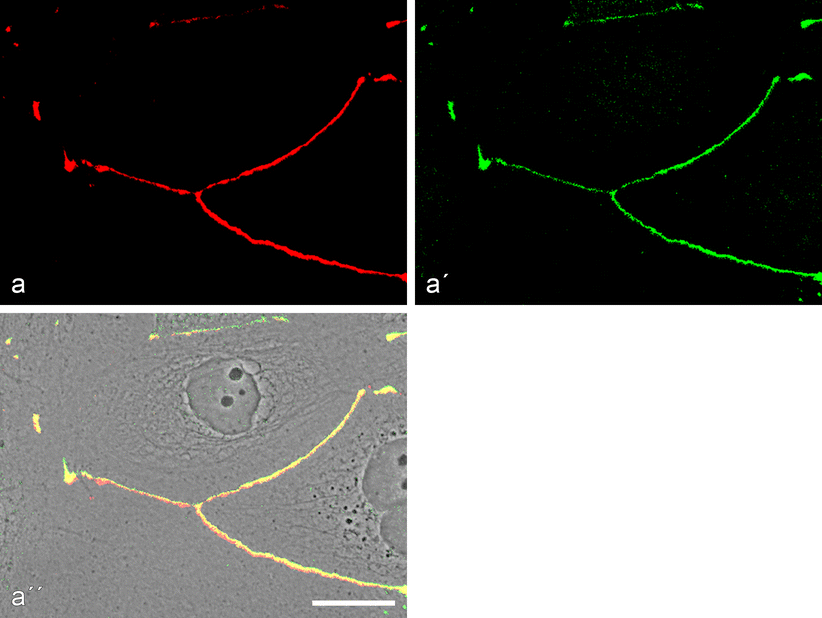

Supplement: Supplementary file 17 — Double-label, confocal laser-scanning immunofluorescence microscopy showing monolayer culture MCF-7 cells (2 days after trypsin-dissociation and re-seeding), double-stained with antibodies against striatin (a, red; mAb m) and rabbit antibodies against occludin (a', green; rb Ab), in a'' on the background of the phase contrast picture. Note that the process of reformation of cell-cell junctions, i.e. of adherens junctions and tight junctions, is not yet complete (e.g., in the upper portion of the picture) but that here both processes, the reformations of a tight junction zonula occludens (occludin) and that of a zonula adhaerens (striatin), are coordinated in time and space, as indicated by the extensive colocalization (yellow merger colour) of both marker proteins in extended junctional structures and in small, isolated intercepts which appear as individual dots or fasciae on plasma membranes or elsewhere in the cytoplasm (e.g. in the upper left region). Bar 20 μm (GIF 96 kb) [file 441_2014_2053_Fig19_ESM.gif]

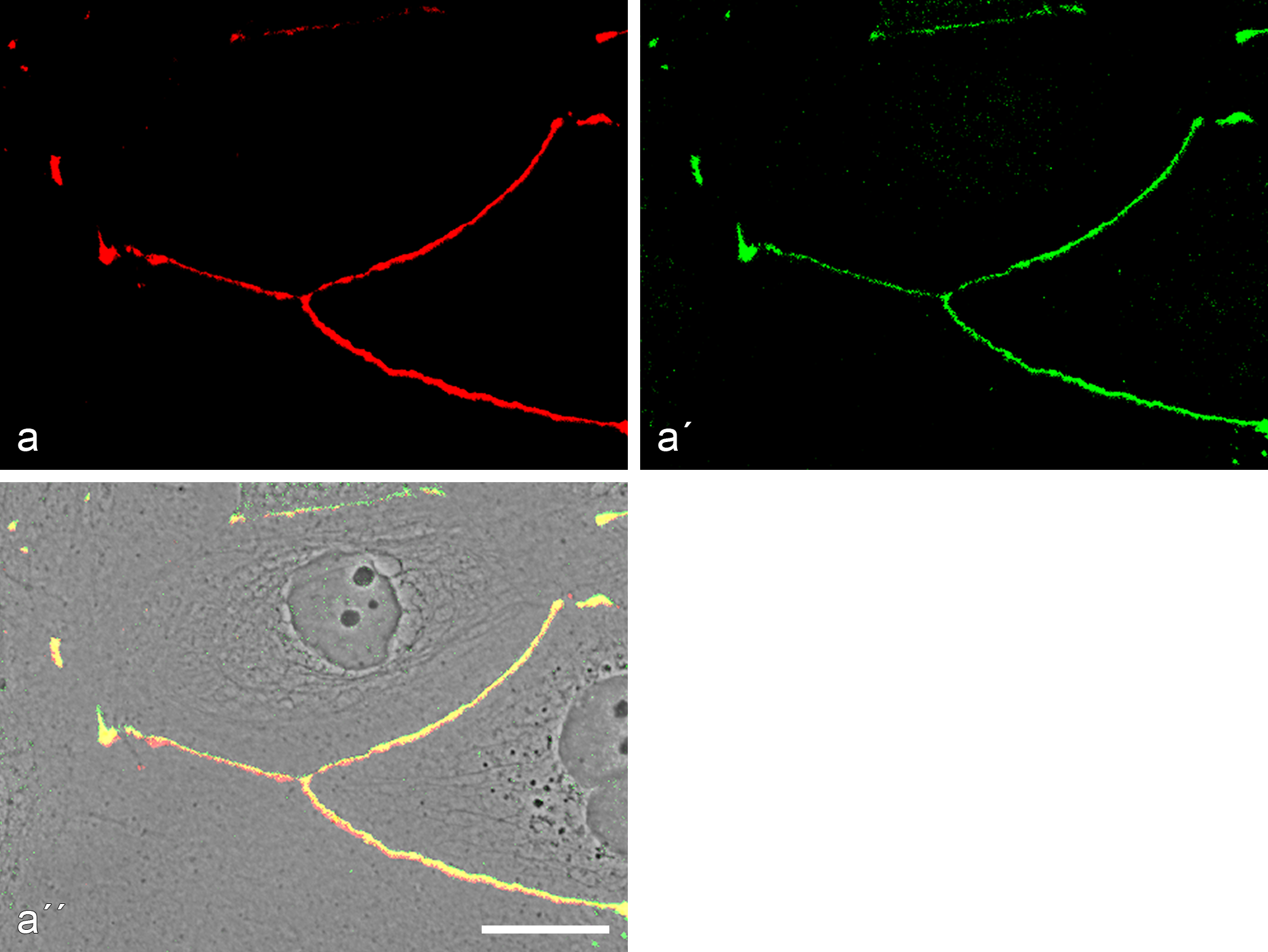

Supplement: Supplementary file 18 — High Resolution (TIFF 1050 kb) [file 441_2014_2053_MOESM9_ESM.tif]

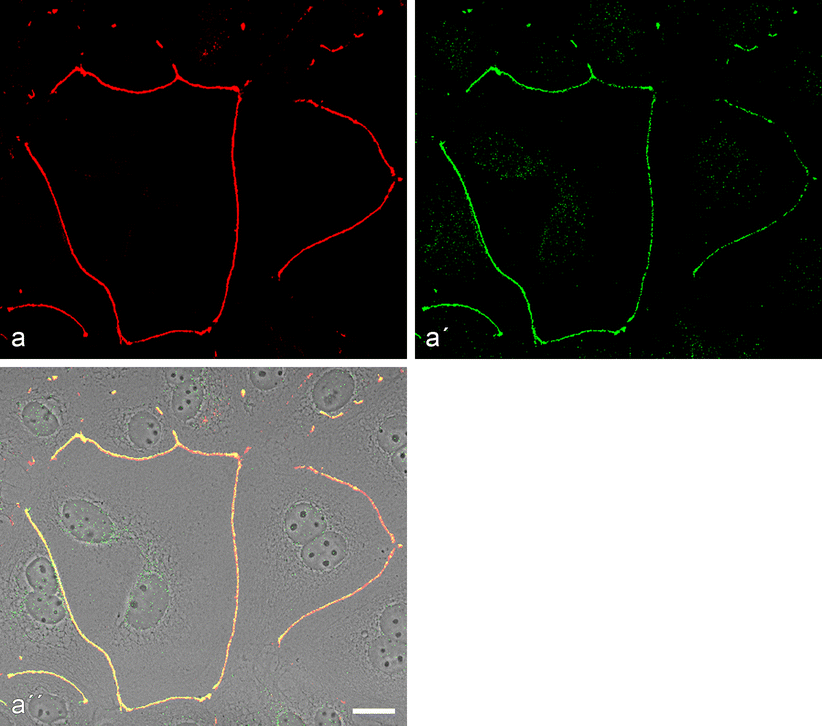

Supplement: Supplementary file 19 — Micrograph showing another region of the MCF-7 cell culture immunostaining preparation presented in the preceeding figure. Note here that during the reformation of the cell-cell contact regions of associated tight and adherens junctions (zonulae occludentes and zonulae adhaerentes) extended cell-cell contact regions with merger colour (yellow) are seen as well as smaller, sometimes tiny structures which are characterized by yellow merger colour, positive for markers of both tight and adherens junctions of widely different lengths. These can occur not only near plasma membranes but also deep in the cytoplasm, partly even in the perinuclear region (e.g., in the upper marginal part of the picture). Bar 20 μm (GIF 126 kb) [file 441_2014_2053_Fig20_ESM.gif]

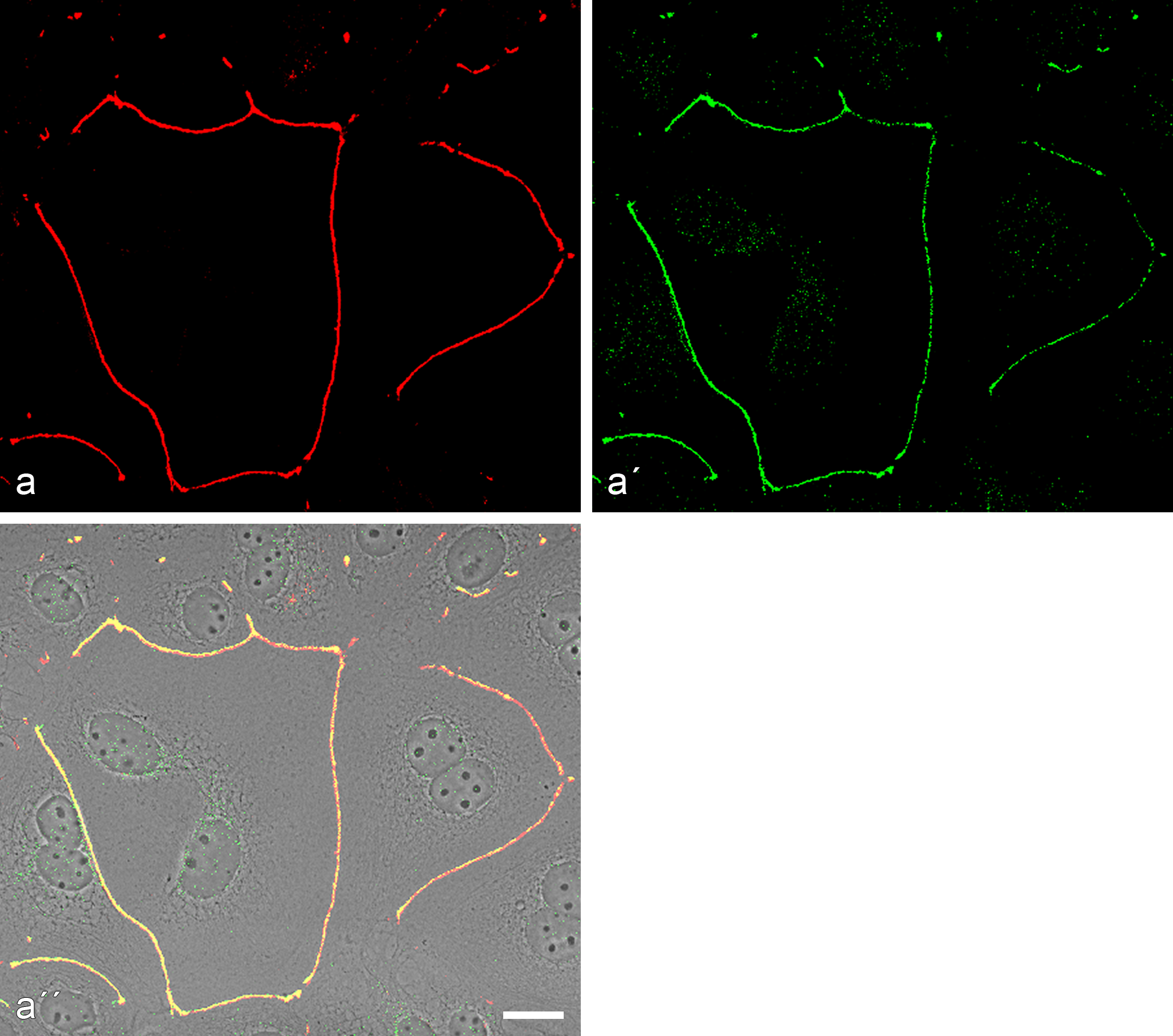

Supplement: Supplementary file 20 — High Resolution (TIFF 1537 kb) [file 441_2014_2053_MOESM10_ESM.tif]
