# Supplementary material for: Striatins as plaque molecules of zonulae adhaerentes in simple epithelia, of tessellate junctions in stratified epithelia, of cardiac composite junctions and of various size classes of lateral adherens junctions in cultures of epithelia- and carcinoma-derived cells
Source: Cell Tissue Res. 2014 Dec 12;359(3):779–97. doi: 10.1007/s00441-014-2053-z (PMC4341017; doi:10.1007/s00441-014-2053-z)
Supplement: Supplementary file 21 — Primary antibodies (DOC 79 kb) [file 441_2014_2053_MOESM11_ESM.doc]

**Table S1 Primary Antibodies**

Abbreviations: As - antiserum or IgGs prepared therefrom; mAb - monoclonal antibody; m - mouse; rb - rabbit; gp - guinea pig

| **Antigen** | **Antibody type** | **Source** |
| --- | --- | --- |
| **Transmembrane Glycoproteins and Proteins** | |  |
| E-cadherin | a) mAb, m  b) mAb, m  c) mAb, rb (EP700Y)  d) As, rb | BD Biosciences Pharmingen (Heidelberg, Germany)  Transduction Laboratories ((Lexington, KY, USA)  Epitomics Inc. (Burlingham, CA, USA)  Epitomics |
| N-cadherin | a) mAb, m  b) mAb, m  c) As, rb | BD Biosciences  Transduction Laboratories  QED Biosciences Inc. (San Diego, CA, USA) |
| P-cadherin | a) mAb, m  b) mAb, m | BD Biosciences  Transduction Laboratories |
| VE-cadherin | a) mAb, m (BV9)  b) mAb, m (BV9)  c) As, rb | Gift of E. Dejana (University of Milan, Italy)  Progen Biotechnik (Heidelberg, Germany)  Cayman Chemical Company (Ann Arbor, MI, USA) |
| Cadherin 11 | a) mAb, m  b) mAb, m  c) As, rb | Zymed Laboratories (now Life Technologies; Darmstadt, Germany)  Invitrogen (now Life Technologies)  Zymed Laboratories |
| Desmoglein 1 | mAb, m (P23) | Progen Biotechnik |
| Desmoglein 2 | a) mAb, m (10G11)  b) mAb, m (G96)  c) mAb, m (G129)  d) mAb, m  e) As, rb  f) As, rb (rb 5)  g) As, gp | Progen Biotechnik  Progen Biotechnik  Progen Biotechnik  Zytomed Systems (Berlin, Germany)  Progen Biotechnik  Progen Biotechnik  Progen Biotechnik |
| Desmoglein 1 + 2 | mAb (DG 3.10) | Progen Biotechnik |
| Desmoglein 3 | mAb, m (G194) | Progen Biotechnik |
| Desmoglein 4 | As, gp | Progen Biotechnik |
| Desmocollin 1 | mAb, m (U100) | Progen Biotechnik |
| Desmocollin 2 | f) As, rb (rb 5)  e) As, gp | Progen Biotechnik  Progen Biotechnik |
| Desmocollin 3 | mAb, m (U114) | Progen Biotechnik |
| Protein PERP | a) mAb m (26.3.30)  b) mAb, m (8.2.9)  c) mAb, m (26.2.22)  d) As, gp (PERP-1A-4B) | Progen Biotechnik  Progen Biotechnik  Progen Biotechnik  Progen Biotechnik |
| Occludin | a) mAb, m  b) mAb, rat5 (MOC37) | Invitrogen  Zymed Laboratories |
| Claudin-1 | a) mAb, m  b) As, rb | Invitrogen  Invitrogen |
| Claudin-4 | As, rb | Invitrogen |

| **Antigen** | **Antibody type** | **Source** | |
| --- | --- | --- | --- |
| **Plaque Proteins** |  |  | |
| α-Catenin | a) mAb, m  b) As, rb | Zymed Laboratories  Sigma (St. Louis, MO, USA) | |
| β-Catenin | a) mAb, m  b) As, rb | BD Biosciences  Sigma (St. Louis, MO, USA) | |
| Plakoglobin | a) mAb, m (11E4)  b) mAb, m (PG 5.1)  c)As, gp | Gift of M.J. Wheelock (University of Nebraska, Omaha, NE, USA)  Progen Biotechnik  Progen Biotechnik | |
| Protein p120 | a) mAb, m  b) As, rb | BD Biosciences  Sigma | |
| Protein p0071 | a) mAb, m  b) As, gp | Progen Biotechnik  Progen Biotechnik | |
| Protein ARVCF | a) mAb, m  b) As, gp | Gift of I. Hofmann (German Cancer Research Center, Heidelberg, Germany)  Progen Biotechnik |  |
| Plakophilin-1 | a) mAb, m (PP1-5C2)  b) As, gp | Progen Biotechnik  Progen Biotechnik |  |
| Plakophilin-2 | a) mAb, m (Pkp2-519)  b) mAb, m (PP2/62,  PP2/86, PP2/150)  c) As, gp | Progen Biotechnik  Progen Biotechnik  Progen Biotechnik |  |
| Plakophilin-3 | a) mAb, m (PKP3-270)  b) As, gp | Progen Biotechnik  Progen Biotechnik |  |
| Desmoplakin | a) mAb, m (DP-2.15, DP‑2.17, DP-2.20)  b) As, gp | Progen Biotechnik  Progen Biotechnik |  |
| Protein ZO-1 | a) mAb, m  b) As, rb | Zymed Laboratories  Zymed Laboratories |  |
| Protein ZO-2 | As, rb | Zymed Laboratories |  |
| Protein Myozap | a) mAb, m (517.67)  b) As, gp | Progen Biotechnik  Progen Biotechnik |  |
| Striatin(s) | a) mAb, m (610838)  b) As, rb  c) As, rb | BD Transduction Laboratories  Millipore (Schwalbach, Germany)  Sigma |  |
| Protein LUMA | a) mAb, m (E-1)  b) mAb, m (F-3)  c) As, gp (2A)  d) As, gp (3A)  e) As, gp (6B) | Santa Cruz Biotechnology (Santa Cruz, CA, USA)  Santa Cruz Biotechnology  Progen Biotechnik; cf. Franke et al. 2014  Progen Biotechnik; cf. Franke et al. 2014  Progen Biotechnik; cf. Franke et al. 2014 |  |
| Plectin | a) As, gp  b) mAb, m | Progen Biotechnik  BD Biosciences |  |

| **Antigen** | **Antibody type** | **Source** | |
| --- | --- | --- | --- |
| **Intermediate-sized Filament Proteins** | | |  |
| Desmin | mAb, m | DAKO (Hamburg, Germany) |  |
| Gliafilament Protein | a) mAb, m  b) As, gp | Progen Biotechnik  Progen Biotechnik |  |
| Most keratins (“pan-keratin”) | mAb, m (Lu5) | Progen Biotechnik |  |
| Keratin 18 | mAb, m (Ks18.04) | Progen Biotechnik |  |
| Keratin 8 | mAb, m (Ks8-17.2) | Progen Biotechnik |  |
| Keratins 8 and 18 | As, gp | Progen Biotechnik |  |
| Vimentin | a) mAb, m (3B4)  b) mAb, m (V9)  c) As, gp  (bVim A+B 06/10) | Progen Biotechnik  Progen Biotechnik  Progen Biotechnik |  |
| **Other Cytoskeletal and Contractile Proteins** | | |  |
| Non-muscle β-, γ-Actin | mAb, m | Sigma |  |
| Smooth muscle α-Actin | mAb, m (ASM-1) | Progen Biotechnik |  |
| Cardiac/embryonic α-Actin | mAb, m (AC1-20.4.2) | Progen Biotechnik |  |
| α-Actinin | a) mAb, m  b) As, rb  c) mAb, m | Sigma  Sigma  Gift of D. Fürst (University of Bonn, Germany) |  |
| l/s-Afadin | As, rb | Sigma |  |
| Ankyrin G | a) mAb, m (4G3F8)  b) As, goat | Life Technologies  Gift of V. Bennett (Duke University, Durham, NC, USA) and P.J. Mohler (Ohio State University, Columbus, OH, USA); cf. Makara et al. 2014 |  |
